# Extracellular Vesicle Capture by AnTibody of CHoice and Enzymatic Release (EV‐CATCHER): A customizable purification assay designed for small‐RNA biomarker identification and evaluation of circulating small‐EVs

**DOI:** 10.1002/jev2.12110

**Published:** 2021-06-03

**Authors:** Megan I. Mitchell, Iddo Z. Ben‐Dov, Christina Liu, Kenny Ye, Kar Chow, Yael Kramer, Anju Gangadharan, Steven Park, Sean Fitzgerald, Andrew Ramnauth, David S. Perlin, Michele Donato, Emily Bhoy, Ehsan Manouchehri Doulabi, Michael Poulos, Masood Kamali‐Moghaddam, Olivier Loudig

**Affiliations:** ^1^ Center for Discovery and Innovation Hackensack Meridian Health Nutley New Jersey USA; ^2^ Laboratory of Medical Transcriptomics Hadassah‐Hebrew University Medical Center Jerusalem Israel; ^3^ Department of Epidemiology and Population Health Albert Einstein College of Medicine Bronx New York USA; ^4^ Biorepository Hackensack University Medical Center Hackensack New Jersey USA; ^5^ Department of Pathology and Laboratory Medicine Weill Cornell Medicine New York USA; ^6^ Department of Immunology, Genetics and Pathology Science for Life Laboratory Uppsala University Uppsala Sweden

**Keywords:** exosome purification, extracellular vesicles, micro‐RNA profiling, sequencing, TEM

## Abstract

Circulating nucleic acids, encapsulated within small extracellular vesicles (EVs), provide a remote cellular snapshot of biomarkers derived from diseased tissues, however selective isolation is critical. Current laboratory‐based purification techniques rely on the physical properties of small‐EVs rather than their inherited cellular fingerprints. We established a highly‐selective purification assay, termed EV‐CATCHER, initially designed for high‐throughput analysis of low‐abundance small‐RNA cargos by next‐generation sequencing. We demonstrated its selectivity by specifically isolating and sequencing small‐RNAs from mouse small‐EVs spiked into human plasma. Western blotting, nanoparticle tracking, and transmission electron microscopy were used to validate and quantify the capture and release of intact small‐EVs. As proof‐of‐principle for sensitive detection of circulating miRNAs, we compared small‐RNA sequencing data from a subset of small‐EVs serum‐purified with EV‐CATCHER to data from whole serum, using samples from a small cohort of recently hospitalized Covid‐19 patients. We identified and validated, only in small‐EVs, hsa‐miR‐146a and hsa‐miR‐126‐3p to be significantly downregulated with disease severity. Separately, using convalescent sera from recovered Covid‐19 patients with high anti‐spike IgG titers, we confirmed the neutralizing properties, against SARS‐CoV‐2 in vitro, of a subset of small‐EVs serum‐purified by EV‐CATCHER, as initially observed with ultracentrifuged small‐EVs. Altogether our data highlight the sensitivity and versatility of EV‐CATCHER.

## Graphical abstract

‘A customizable, low background, high‐affinity assay for specific immuno‐capture and release of circulating small extracellular vesicles prior to small‐RNA sequencing for identification of miRNA biomarkers or in‐vitro evaluation: The EV‐CATCHER assay (Extracellular Vesicle Capture by AnTibody of CHoice and Enzymatic Release)’.

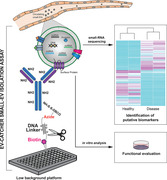

## 1 INTRODUCTION

Nucleic acids released by cells during infection, inflammation, cancer, and other physiological and pathological processes can be found circulating in human blood and represent potentially powerful biomarkers of disease (Mandel & Metais, 1948; Schwarzenbach & Hoon, 2011). Technologies detecting circulating cell‐free genomic tumour DNA mutations are, for example, showing great promise to evaluate treatment response and presence of residual disease in cancer (Cabel et al., 2018; Cescon et al., 2020; O'Leary et al., 2018; Reece et al., 2019). While large single‐stranded RNA transcripts are degraded in circulation, small‐RNA molecules, and in particular microRNAs (miRNA; ∼22 nt long), remain intact and can be detected and measured to reflect pathological processes. MiRNAs are master transcriptional regulators that modulate the activity of specific mRNA targets and play important roles in a wide range of normal and pathological processes (Arroyo et al., 2011; Cortez et al., 2011; Larrea et al., 2016; LeBleu & Kalluri, 2020; Shah et al., 2018; Zlotorynski, 2019). Despite significant progress in detection of circulating miRNAs, the discovery of disease‐related miRNA biomarkers has been hindered by the low representation of these molecules within the large pool of circulating miRNAs, which originate from diverse cellular and tissue sources. Such limitation requires development of molecular assays with greater detection specificity and sensitivity. To address this issue, studies are now focused on the analysis of miRNA cargos encapsulated within circulating extracellular vesicles (EVs), rather than total RNA purified from whole blood, serum, or plasma (Mitchell et al., 2008; Rohan et al., 2019). For example, a population of small‐EVs, namely exosomes, are showing great promise due to their active release by most cell types into the microenvironment and the circulation. These small‐EVs have been shown to participate in intercellular communications via targeted cellular uptake and cytoplasmic release of their miRNAs cargos, which have been implicated in the re‐programming of recipient cells (Fan et al., 2018; O'Brien et al., 2020; Simons & Raposo, 2009; Wang et al., 2019; Zhang et al., 2015). Studies on exosomes have provided the basis for small‐EV purification but also highlighted the strong potential of their unique small‐RNA cargos for evaluation as circulating biomarkers from most biofluids (Armstrong et al., 2015; Cheng et al., 2019; Li et al., 2015; Rodríguez et al., 2017; Srinivasan et al., 2019). These specific small‐EVs can be differentiated by both their unique size range (30‐150 nm in diameter) and their biogenesis (Colombo et al., 2014). Produced via the endosomal pathway, exosomes are packaged with unique cellular material (RNA, gDNA, and proteins) and specifically enriched in membrane‐bound tetraspanins CD9, CD63, CD81, CD37 and CD82 (Andreu & Yáñez‐Mó, 2014; Colombo et al., 2014). These surface proteins can be targeted by immuno‐purification for global small‐EV analyses (Colombo et al., 2014; Andreu & Yáñez‐Mó, 2014). However, during biogenesis these small‐EVs also inherit surface proteins from their cell of origin. Customizable antibody‐based purification assays targeting these cellular fingerprints have potential to improve detection of potentially unique and clinically relevant biomarkers (Larssen et al., 2017; Mathivanan & Simpson, 2009; Wu et al., 2019).

Although major advances have been made over the last few years with the development of microfluidic‐based systems for purification of exosomes and other types of small‐EVs, very few have reached the commercial market and thus laboratory‐based techniques remain the gold standard for their purification and evaluation from biofluids. To date, four principal circulating exosomes/small EVs purification approaches have been developed, with a majority of them providing an averaged biomarker evaluation via a bulk small‐EV selection (Ludwig et al., 2018; Patel et al., 2019; Yu et al., 2018). Three classical approaches to purifying small‐EVs include ultracentrifugation, precipitation, and size‐exclusion (Ludwig et al., 2018; Macías et al., 2019; Patel et al., 2019; Yu et al., 2018). Although these methods result in pure preparations (with caveats for precipitation‐based methods), all of them produce a mix of small‐EV sub‐populations released into the circulation by various cell types and tissues, confounding and limiting subsequent analyses. The fourth method employs magnetic beads coated with monoclonal antibodies for immuno‐purification by selection of known surface biomarkers (Larssen et al., 2017; Löf et al., 2017; Pugholm et al., 2015). Although streptavidin magnetic‐beads may allow for a customizable biotinylated antibody‐based selection, this customization requires optimization, is highly dependent on the quality (surface polymer) and the chemistry (streptavidin and carboxylic acid coating) of the magnetic beads. The design of an easily customizable low background antibody‐selection assay may not only allow for more sensitive purification of small‐EV subpopulations, but may also provide a versatile tool for the selection of cell‐specific surface biomarkers and in turn fine‐tuned identification of unique disease biomarkers circulating in biofluids (Williams et al., 2013). Finally, and most importantly, although a few low pH elution buffers have been described in the literature (Chen et al., 2020; Hisey et al., 2018; Hong et al., 2014), very few manufacturers provide such buffers for release of the covalently‐bound antibodies and intact release of selected small‐EVs for downstream in vitro analyses (Hardin et al., 2018; Patel et al., 2019; Peterson et al., 2015; Theodoraki et al., 2018; Velandia‐Romero et al., 2020). A recent immunoaffinity‐based exosome isolation assay (exo‐PLA) addressed many of these issues by using an enzymatically degradable DNA linker between the exosome‐binding antibody and the magnetic bead, permitting release of intact exosomes (Löf et al., 2017). However, our initial evaluations strongly indicated that magnetic beads, used with this approach, could non‐specifically bind small‐RNAs to a degree significantly limiting detection of low‐abundance miRNA cargos.

In addition to representing a valuable source of biomarkers, circulating exosomes/small‐EVs are also key players in multiple biological processes, particularly in the immune system (Barros et al., 2018; Burkova et al., 2019; Gutzeit et al., 2014; Huang et al., 2018; Mallegol et al., 2007; Smith et al., 2017). Notably, recent studies have suggested that exosomes, in particular, participate in antigen presentation to the immune system (Mallegol et al., 2007; Smith et al., 2017) and may mediate immune defense during viral infection (Barros et al., 2018; Gutzeit et al., 2014). The mechanistic role that exosomes play in immunity against well studied viruses (HIV, EBV, Ebola) has previously been evaluated (Mukhamedova et al., 2019; Pleet et al., 2016; Vallhov et al., 2011). However, few studies have evaluated the potential roles or implications of circulating small‐EVs for SARS‐CoV‐2 infections. Our health care network (Hackensack Meridian Health (HMH)), which includes 17 hospitals, was located within the U.S. epicentre of the SARS‐CoV‐2 pandemic where nearly half of all New Jersey Covid‐19 patients were hospitalized. This allowed us to gain access to two different biorepository‐based cohorts of serum samples from Covid‐19 patients for use in proof‐of principle pilot studies where we evaluated our customizable antibody‐based purification assay, which we designed for small‐RNA cargo analysis and intact release of functional small‐EVs purified from clinical specimens.

In this methodology report we present a new small‐EV purification assay, termed Extracellular Vesicle Capture by AnTibody of CHoice and Enzymatic Release or EV‐CATCHER, which allows for customizable selection and release of immuno‐purified small‐EVs. We describe its step‐by‐step design and thorough evaluation by comparison to 11 different small‐EV purification methods. As proof‐of‐principle purification of circulating small‐EVs with EV‐CATCHER and for comparison to existing methods, we chose antibodies targeting tetraspanins expressed on small‐EVs (anti‐CD63, ‐CD9, ‐CD81 antibodies) for our analyses. Using EV‐CATCHER in combination with our highly‐sensitive small‐RNA cDNA library preparation protocol (Loudig et al., 2017, 2018), designed for high‐throughput small‐RNA sequencing, we purified circulating small‐EVs of sera collected from Covid‐19 patients at the time of their hospitalization (first set of clinical specimens) and identified differentially expressed miRNAs associated with severity of the disease, otherwise not significantly detectable in whole sera. Additionally, to evaluate integrity of the small‐EVs enzymatically released by EV‐CATCHER, we confirmed the neutralizing activity of small‐EVs purified from sera of recovered Covid‐19 individuals (Second set of clinical specimens), which we had unexpectedly and initially identified by using ultracentrifuged small‐EVs from convalescent donors with high anti‐spike IgG titers.

## 2 MATERIALS AND METHODS

### 2.1 Experimental and clinical specimens

Human MCF‐7 and mouse RAWS 264.7 small‐EVs obtained from tissue culture media and purified by ultracentrifugation were purchased from System Biosciences (Cat#: EXOP‐100A‐1 and EXOP‐165A‐1 respectively). MCF‐7 small‐EVs (30‐150 nm) for protein titration experiments were obtained by ultracentrifugation of media. Briefly, 60 ml of tissue culture media (comprised of DMEM (Gibco, #10566016) supplemented with 10% small‐EV depleted FBS (Gibco, #A2720801) and 1% PenStrep (Gibco, #15140122)), obtained from ∼80% confluent MCF‐7 cells, was refreshed 48 h before collection. Cells, dead cells and cell debris were removed by centrifugation at 500 × *g* for 5 min, 2,000 × *g* for 10 min, followed by 10,000 × *g* for 30 min, respectively. Ultracentrifugation of small‐EVs from culture media, human plasma, or serum was performed in 10 ml Ultraclear tubes (Beckman Coulter, #344059) in an Optima XE‐90 ultracentrifuge (Beckman Coulter) for 2 h at 100,000 × *g* at 4°C. Pellets were washed with 1× PBS (Gibco, #10010023) and then subjected to a second round of ultracentrifugation for 2 h at 100,000 × *g* at 4°C and final small‐EV pellets were resuspended in 50 μl 1× PBS. Serum specimens from SARS‐CoV‐2 infected patients, at the time of their hospitalization (first sample set used for pilot identification of differentially expressed miRNAs between mild and severe Covid‐19 disease), were collected at Hackensack University Medical Center (HUMC) under IRB#Pro2018‐1022 within the HMH Network. Serum samples were processed at the Biorepository (BioR) and stored as 500 μl aliquots at ‐80°C. Upon submission of an internal proposal (Proposal# BioRCOV4) and approval, 13 serum samples from patients who did not require mechanical ventilation and 17 samples from mechanically ventilated patients with moderate to severe ARDS were collected and both whole sera and EV‐CATCHER purified small‐EVs were subjected to RNA extraction. Original convalescent serum specimens (second sample set used for evaluation of viral neutralization by convalescent small‐EVs) were collected from recently recovered Covid‐19 individuals enrolled in the convalescent plasma study lead by Dr. Michele Donato (IRB#Pro2020‐0375/0378). Excess samples were transferred to the HMH BioR, after being de‐identified and were provided to the Center for Discovery and Innovation (CDI) after review and approval by the Covid‐19 Research Review Committee (proposal COVID‐19#102). Provided samples were stored as 1,000 μl aliquots at ‐80°C. Enzyme‐linked immunosorbent assays (ELISA), using the Receptor Binding Domain (RBD) of SARS‐CoV‐2 spike protein as antigen, developed and optimized in the laboratory of Dr. David Perlin to identify high and low anti‐spike IgG serum titers were initially performed. Sera samples were then stratified as either high IgG (titer > 10,000) or low IgG (titer Below Limit of Quantification (BLQ)) titers. Three sera samples from high IgG and 3 from BLQ IgG convalescent individuals were subjected to CD63 purification using the EV‐CATCHER assay and evaluated for neutralization potential of their circulating small‐EVs. Two samples (one high and one BLQ) were ultracentrifuged for evaluation of small‐EVs from whole serum.

### 2.2 Small‐EV Capture by AnTibody of CHoice and Enzymatic Release (EV‐CATCHER) assay

The release protocol described by Löf et al. (2017) was further optimized and modified as follows (Löf et al., 2017); HPLC‐purified uracilated oligonucleotides (Integrative DNA Technology) for the 5′‐Azide (5′Az‐AAAAACGAUUCGAGAACGUGACUGCCAUGCCAGCUCGUACUAU CGAA) and 3′‐Biotin (5′Bio‐CGAUAGUACGAGCUGGCAUGGCAGUCACGUUCUCGAA UCGUUUU), adapted from Löf et al. (2017) were resuspended in RNase‐free water at a concentration of 250 ng/μl. Equimolar amounts (1:1 ratios) of each oligo were annealed (90°C for 2 min, 90‐42°C for 40 min, 42°C for 120 min) in 1× RNA annealing buffer (60 mM KCl, 6 mM HEPES (pH 7.5), 0.2 mM MgCl_2_), prior to separation on a 15% non‐denaturing polyacrylamide (PAGE) gel (0.5× TBE (ThermoFisher, #15581044) at 450 volts for 90 min). The double stranded (ds) DNA linker was visualized on a blue light box with SYBR Gold dye (ThermoFisher, #S11494), excised, centrifugally crushed using a gel breaker tube (IST Engineering, #3388‐100) and resuspended in 400 mM NaCl and shaken overnight (O/N) on a thermomixer set to 4°C and 1,100 RPM. The solution was filtered, and the dsDNA linker was purified using the QIAEX^®^ II gel extraction kit (Qiagen, #20021) according to manufacturer instructions. Purified dsDNA linker was evaluated on a NanoDrop 2000 and diluted to 250 ng/μl. Antibodies (1 mg/ml) used for exosome pulls (anti‐CD63 (Abcam, #ab59479), anti‐CD81 (Abcam, #ab233692) and anti‐CD9 (Abcam, #ab263023)) were activated using 5 μl of freshly prepared 4 mM DBCO‐S‐S‐NHS ester (Sigma Aldrich, #761532) and incubated for 30 min at room temperature (RT) in the dark, reactions were stopped by adding 2.5 μl of 1 M Tris‐Cl (pH 8.0) at RT for 5 min in the dark. DBCO‐activated antibodies were desalted onto pre‐equilibrated Zeba desalting columns (ThermoFisher, #89882) by incubation for 1 min and centrifugation at 1500 × *g* for 2 min. Antibodies were quantified on a Nanodrop 2000 instrument using protein A280 and antibody‐dsDNA (Ab‐dsDNA) stock solutions were prepared by conjugating 50 μg of activated antibody with 25 μg of purified DNA linker, O/N at 4°C on a rotator. Validation of Ab‐dsDNA binding was performed by PAGE where the Ab‐dsDNA (1 μg) product was run under non‐denaturing and non‐reducing conditions, followed by Coomassie (Bio‐Rad #1610786) staining to visualize the shift in Ab‐dsDNA migration. The next day, Ab‐dsDNA conjugates were bound to streptavidin coated 96‐well plates (Pierce, #15120). Either single anti‐CD63 antibody (1 μg) or a combination of anti‐CD63, ‐CD81 and ‐CD9 (1 μg of each antibody) (linker bound) was added to single wells in 100 μl 1x PBS. Streptavidin coated 96‐well plates with Ab‐dsDNA conjugates were placed on a plate shaker at 300 RPM at 4°C, for 8 h to allow for binding to the plate. Solutions were carefully removed, and wells were washed three times with cold 1× PBS solution, prior to addition of RNase‐A (12.5 μg/ml) treated samples (100 μl). Plates were sealed using microAMP optical adhesive film (Applied Biosystems, #4311971) and placed on a shaker at 300 RPM at 4°C, O/N. Samples were carefully removed, wells were washed 3 times with cold 1x PBS and 100 μl of freshly prepared uracil glycosylase (UNG) enzyme (ThermoFisher, #EN0362) in 1x PBS (1x UNG buffer (200 mM Tris‐Cl (pH 8.0), 10 mM EDTA and 100 mM NaCl), with 1 unit of enzyme) was added to each well. Plates were incubated at 37°C for 2 h on a shaker at 300 RPM for UNG digest of the dsDNA linker, and small‐EVs were recovered in this solution for further characterization and downstream analyses.

### 2.3 Small‐EV purification comparisons

Non‐specific capture of ath‐miR‐159a and small‐EVs (10μg) from MCF‐7 tissue cultured human breast cancer cells lines (Abcam Cat#ab239691) was performed by comparison of our optimized EV‐CATCHER assay to 10 different globally distributed commercial kits, which included: 1‐ Exosome Isolation CD63 kit from Miltenyi Biotec (Cat#130‐110‐918); 2‐ Mojosort Magnetic beads from Biolegends (Cat#480016); 3‐ MagCapture Tim 4 Exosome isolation kit from Fujifilm (Cat#293‐77601); 4‐ Dynabeads MyOne T1 Carboxylic Acid beads from Thermofisher (Cat#65011); 5‐ ExoCap CD63+ from MBL International (Cat#MEX‐SA123); 6‐ Dynabeads Streptavidin MyOne T1 beads from ThermoFisher (Cat#10606D); 7‐ Exo‐Flow32 CD63 IP exosome purification kit from System Biosciences (SBI) (Cat#EXOFLOW32A‐CD63); 8‐ ExoEasy exosome purification kit from Qiagen (Cat#76064); 9‐ Plasma/serum exosome purification kit from Nörgen Biotek (Cat#57400); 10‐ ExoQuick purification reagent from SBI (Cat#EXOQ5TM‐1); and Ultracentrifugation (Method#11) for sedimentation of small‐EVs as described in the ‘Experimental and clinical specimens’ section above. For all commercial kits, we followed manufacturer's instruction to purify small‐EVs directly, or by binding the same anti‐CD63 antibody (Mojosort, Dynabeads streptavidin and carboxyl beads, and ExoCap) used with EV‐CATCHER. For evaluation of non‐specific small‐RNA binding to magnetic‐beads (Methods 2, 4, 5 and 6), we used 100pg of ath‐miR‐159a (IDT) spiked into 100μl 1× PBS and the RNA extractions were performed after three successive washes using the miRNeasy serum/plasma kit from Qiagen (Cat#217184). The non‐specific binding of 100pg ath‐miR‐159a spiked into 100 μl of human serum (Sigma Cat#H4522) was evaluated for all methods where all magnetic beads were conjugated to CD63 (Commercial kits #1,2,4,5,6 and 7), or Tim 4 (commercial kit # 3), following manufacturer's instruction, final elutions using kit provided elution buffers (ExoFlow elution buffer and including 1% formic acid pH3 solution), and RNA extraction using the miRNeasy serum/plasma kit from Qiagen. Non‐specific binding of small‐EVs to magnetic beads was also evaluated using small EVs from MCF‐7 cells (SBI, Cat#EXOP‐100A‐1) using unconjugated magnetic beads (Commercial kits# 2, 4, 5 and 6).

### 2.4 RNA extractions

Whole sera, whole plasma, and small‐EVs isolated from biofluids were subjected to total RNA extraction using the miRNeasy Serum/Plasma kit (Qiagen, Cat#217184) according to manufacturer's instructions with some modifications to improve total RNA yield. Briefly, QIAzol was added to 100 μl of whole plasma, whole serum, or small‐EVs purified from Biofluids, vortexed and incubated at RT for 5 min, after which chloroform was added to each sample. Samples were vortexed again and incubated at RT for 5 min. Samples were centrifuged at 12,000 × *g*, at 4⁰C for 15 min and the upper aqueous phase of each sample was carefully removed and transferred into new siliconized tubes, to which 100% ethanol was added. Samples were incubated on ice for 40 min prior to column purification. The clear upper phase was then passed twice through supplied RNeasy minElute columns, washed with RPE, and ice cold 80% ethanol. Columns were spun to remove residual ethanol and total RNA was eluted with 50 μl of RNase‐free water. Samples were then speed‐vacuumed to 10 μl for small‐RNA sequencing or to 20 μl for quantitative real‐time PCR (RT‐qPCR). For cDNA small‐RNA library preparation and next‐generation sequencing, 3 ng of size primers (1.5 ng ‐ 19 nt and 1.5 ng ‐ 24 nt; (Loudig et al., 2018)) was spiked into each RNA extraction. For RT‐qPCR experiments, 1 pg of ath‐miR‐159a (IDT, rUrUrUGGArUrUGAAGGGAGCrUCrUA) was added to QIAzol prior to RNA extractions from MCF‐7 and clinical specimens (mild vs. severe Covid‐19 samples) prior to RNA separation and column purification and used as an exogenous technical normalization control.

### 2.5 Western blot analyses

Western blot analyses were conducted to evaluate presence of surface protein biomarkers from purified small‐EV fractions. Purified small‐EVs were lysed and denatured in 1× Laemmli buffer (Bio‐Rad, #161‐0747) containing 355 mM β‐mercaptoethanol, and heated at 95°C for 5 min. Denatured lysates were pulse centrifuged and separated on 4%–12% polyacrylamide precast mini‐PROTEAN TGX gel (Bio‐Rad, # 4561086) by sodium dodecyl sulphate polyacrylamide gel electrophoresis (SDS‐PAGE). Five microliters of Precision Plus Protein Dual Xtra Prestained Protein Standard (Bio‐Rad, #1610377) was loaded and used for gel orientation and determination of molecular weights of separated proteins. Samples were loaded and gels were run at 100 V and 400 mA for 90 min (Power Pac 300, Bio‐Rad) in 1× Tris/Glycine/SDS buffer (Bio‐Rad, #1610732). After the SDS‐PAGE run, proteins were transferred to 0.2 μm polyvinylidene fluoride (PVDF) membranes (Bio‐Rad, #1704156) using a semidry electro‐transfer system (TransBlot Turbo v1.02, Bio‐Rad) for 30 min at 25 V. Membranes were visualized using the stain‐free blot protocol provided on a Chemi‐Doc MP (Bio‐Rad) system to evaluate protein transfer and non‐specific binding was prevented by blocking membranes in EveryBlot blocking buffer (Bio‐Rad, #12010020) for 30 min. Membranes were incubated at 4°C O/N with TBS‐T (1x TBS, pH 6.8, 0.1% Tween20) diluted anti‐mouse primary antibodies (1:1000) targeted against Apolipoprotein B (Novus Biologicals, Cat#MAB4124), Alix (Life Technologies, #MA183977), Albumin (Novus Biologicals, Cat#MAB1455), CD63 (Abcam, Cat#ab59479), or with TBS‐T diluted anti‐rabbit primary antibodies (1:1000) targeted against Apolipoprotein A1 (Novus Biologicals, Cat#MAB36641), CD81 (Abcam, #ab233692), and CD9 (Abcam, #ab263023). Membranes were washed with TBS‐T (3 × 5 min) before incubation in either anti‐mouse or anti‐rabbit IgG horseradish peroxidase conjugated secondary antibodies (1:10000) for 1 h, with gentle agitation at RT. Membranes were washed with TBS‐T (3 × 5 min) before proteins were detected using SuperSignal West Femto Maximum Sensitivity Substrate (Pierce, #34095) and protein bands were visualized using ImageLab 4.0 software on a Chemi‐Doc MP (Bio‐Rad) imaging system.

### 2.6 Transmission electron microscopy

Transmission electron microscopy (TEM) of purified small‐EVs was performed by Charles River Laboratories (Pathology Associates (PAI), Durham NC) and at the analytical imaging facility at the Albert Einstein College of Medicine, Bronx, NY. Briefly, purified small‐EVs were fixed using 2% Glutaraldehyde in phosphate buffer (Electron Microscopy Services, #6536‐05) and stored at 4°C. 300 mesh formvar‐coated grids were inverted onto 20 μl of fixed exosome suspensions for approximately 2 min and wicked dry. Grids were then inverted onto 40 μl of 2% aqueous uranyl acetate for approximately 1 min, and wicked dry. Samples were imaged on a JEOL JEM‐1400+ transmission electron microscope (JEOL Ltd.; Tokyo, Japan) operating at an accelerating voltage of 80 kV. High resolution TIFF images were acquired and saved using an AMT 16 MP digital camera system (Advanced Microscopy Techniques Corp.; Woburn, MA).

### 2.7 Nanoparticle tracking

Size and particle concentration of purified small‐EVs were assessed using a Spectradyne nCS1 instrument. Briefly, 2 μl of purified small‐EVs were loaded onto TS400 microfluidic cartridges (Spectradyne, #TS400), which allows for the analysis of particles between 65–400 nm. Loaded cartridges were primed in the instrument using 0.2 μm filtered buffer (1× PBS containing 1% Tween‐20) and particle acquisition was carried out. Acquired stats files were analysed for particle concentration and size distribution using Spectradyne Viewer software, where peak‐filtered CSD graphs were generated.

### 2.8 Small‐RNA cDNA library preparations

Small‐RNA sequencing from small‐EVs was performed using the cDNA library preparation protocol described by Loudig et al. (2017), with modifications for low input RNA from biofluids and purified small‐EVs (Loudig et al., 2018). For optimization of the sequencing method, titration experiments were performed in our laboratory using decreasing amounts of total RNA extracted from whole serum with as little as 1.5 ng of total RNA (see Figure 4a). For small‐EVs separately purified from Covid‐19 sera, the small‐RNA cDNA library preparation was performed using total RNA recovered from small‐EVs purified from 200 μl of serum (2 wells were prepared for each serum sample (100 μl per well) and pulls were pooled for RNA extraction. Briefly, 15 (each of the Covid‐19 libraries for the first set of serum samples) to 18 (optimization experiments) ligations were set up individually by combining 9.5 μl of total RNA, 8.5 μl of the master‐mix and 1 μl of 50 μM adenylated barcoded 3′ adapter (Integrated DNA Technologies, custom order). A master‐mix was prepared using 0.0052 nM calibrator cocktail (40 μl 10× RNA ligase ‐2 buffer, 120 μl 50% DMSO, and 10 μl calibrator cocktail from (Loudig et al., 2018)). Reactions were heated at 90°C for 1 min, incubated on ice for 2 min, and 1 μl (1/10 diluted) truncated K227Q T4 RNA Ligase 2 (New England Biolabs, #M0351L) was added to each reaction, which were then incubated O/N on ice in a cold room. The next day, ligations were heat inactivated at 90°C for 1 min, and individually precipitated by addition of 1.2 μl of Glycoblue mix (1 μl Glycoblue Co‐precipitant (15 mg/ml; ThermoFisher, #AM9516) in 26 μl 5 M NaCl (ThermoFisher, #AM9579)) and 63 μl of 100% ethanol was added to each tube. Reactions were combined, precipitated on ice for 1 h and centrifuged for 1 h at 14,000 RPM, at 4°C. The pellet was dried and resuspended in 20 μl nuclease‐free water and 20 μl denaturing PAA gel loading solution and separated on a 15% Urea‐PAGE gel. Size marker RNA oligonucleotides (IDT) were used as guide for gel excision. The gel piece was crushed using a gel‐breaker tube (IST Engineering, #3388‐100) and incubated in 400 mM NaCl O/N at 4°C, at 1,100 RPM on a thermomixer. The next day the solution was filtered and precipitated in 100% ethanol on ice for 1 h. RNA pellet was obtained by centrifugation at 14,000 RPM for 1 h at 4°C. The 5′ adapter was added to the resuspended pellet and T4 RNA Ligase 1 (New England Biolabs, #M0204L) was added for 1 h at 37°C. The ligated product was separated on a 12% Urea‐PAGE gel in the presence of 5′ ligated size markers, as guide for size selection. The gel was spun in a gel breaker tube, after which the crushed gel was resuspended in 300 mM NaCl solution with 1 μl 100 μM 3′ PCR primer, and incubated O/N on a thermomixer at 1100 RPM at 4°C. Subsequently, the solution was filtered, precipitated with 100% ethanol, incubated on ice for 1 h and pelleted by centrifugation at 14,000 RPM for 1 h at 4°C. The RNA pellet was resuspended in nuclease free water for reverse transcription (3 μl 5× first strand buffer, 1.5 μl of 0.1 M DTT, and 4.2 μl dNTP Mix (2 mM each; ThermoFisher, #R0241)) with 0.75 μl SuperScript III Reverse Transcriptase (ThermoFisher, #18080‐093) and incubated at 50°C for 30 min. Reverse transcription was deactivated at 95°C for 1 min, followed by addition of 95 μl nuclease‐free water. A pilot PCR reaction was set up (10 μl 10x PCR buffer, 10 μl dNTP Mix (2 mM each), 10 μl cDNA, 67 μl nuclease‐free water, 0.5 μl 3′ PCR primer, 0.5 μl 5′ PCR primer, and 2 μl Titanium Taq DNA Polymerase (Clontech Laboratories, #639208)). 12 μl aliquots were withdrawn at cycles 10, 12, 14, 16, 18, 20 and 22 for analysis on a 2.5% agarose gel, and identification of the optimal PCR amplification cycle. Six PCR reactions were then set up, run for the optimal number of amplification cycles, a portion (10 μl) was evaluated on a 2.5% agarose gel. The remaining solution was combined, precipitated, digested with PmeI for removal of size markers, and separated on a 2.5% gel. The 100 nt PCR library product was excised, purified with QIAquick Gel Extraction Kit (Qiagen, #28704) and quantified using the Qubit dsDNA HS Assay Kit (ThermoFisher, #Q32854). cDNA libraries were then sequenced (single‐read 50 cycles) on a HiSeq 2500 Sequencing System (Illumina, #SY‐401‐2501), after which FASTQ files containing raw sequencing data were processed for adapter trimming and small‐RNA alignment to the hg‐19 genome. Read counts were normalized to total counts and subjected to statistical analyses (see below).

### 2.9 Quantitative PCR experiments

Quantitative PCR (qPCR) experiments were performed using TaqMan microRNA reverse‐transcription kits, TaqMan microRNA master mix PCR kits, and individual TaqMan microRNA assays following manufacturer's instructions (ThermoFisher). For evaluation of non‐specific small‐RNA binding to magnetic beads (Using uncoupled magnetic beads that included Dynabeads Streptavidin (ThermoFisher); ExoCap (MBL international); Dynabeads MyOne Carboxylic Acid (Thermofisher); and Mojosort Magnetic beads (Biolegends)) and our streptavidin coated 96‐well plates, we evaluated the non‐specific binding of one hundred picograms of ath‐miR‐159a RNA oligonucleotide (Integrated DNA Technologies) and performed qPCR reactions of elutions from the different beads after three 1×PBS washes. For optimization experiments we quantified hsa‐miR‐21 (ThermoFisher, Cat#000397) and hsa‐miR‐200c (ThermoFisher, Cat#000505) with RNA extracted from MCF‐7 exosomes (SBI). For validation of differentially expressed miRNAs identified by sequencing of RNA of small‐EVs from Covid‐19 serum specimens, TaqMan miRNA primer assays for hsa‐miR‐126‐3p (Cat#002228), hsa‐miR‐146a (Cat#002163), hsa‐miR‐126‐5p (Cat#000451), hsa‐miR‐205 (Cat#000509) were used for qPCR detection. For these quantifications, one picogram of ath‐miR‐159a was added to the Covid‐19 infected serum specimens prior to RNA extractions and used as a qPCR normalization control. For these qPCR reactions, reverse transcriptions were set up using 10% (2 μl out of 20 μl) of RNA extracted from combined CD63/CD81/CD9 small‐EV purifications from 100 μl of serum. For individual transcript quantifications, 1/3 of the RT reactions (5 μl) was used to set up the triplicate individual qPCR experiments. Quantitative PCR measurements were performed on a Step‐One‐Plus instrument using manufacturer's recommended 96‐well plates and sealing covers. Quantitative qPCR data was transferred to Excel sheet for statistical analyses, as described below.

### 2.10 BSL‐3 tissue culture and neutralization of SARS‐CoV‐2

Vero 76, clone E6 (Vero E6) cells (ATCC, #CRL‐1586), maintained in Eagle's Minimum Essential Medium (EMEM) supplemented with 10% Fetal Bovine Serum (FBS) (Gibco, #26140079) and 1% Penicillin‐Streptomycin‐Amphotericin B (ATCC, #PCS‐999‐002), were seeded in wells of a 96‐well black, clear flat‐bottom culture plate (Corning, #07‐200‐565) at a density of 20,000 cells per well. Once plated, cells were maintained at an atmosphere of 37°C with 5% CO_2_ humidity overnight to allow cells to attach and form a ∼80% confluent monolayer. Prior to treatment and SARS‐CoV‐2 viral infection, cell monolayers were washed with pre‐warmed 1× PBS and 50 μl of fresh media was added. Cell treatments were prepared, wherein, whole sera, small‐EVs purified using CD63 EV‐CATCHER or by ultracentrifugation from convalescent sera (using the second set of Covid‐19 serum samples), were diluted (5 μl in 200 μl (40× dilution) with pre‐warmed growth media. A total of 25 μl of the prepared treatment solutions was added in each wells with 50 μl of pre‐warmed growth media, cells were incubated at an atmosphere of 37°C and 5% CO_2_ humidity for 2 h. After which, small‐EV‐treated Vero E6 cells were inoculated with 25 μl of mNeonGreen (mNG) infectious cDNA clone of SARS‐CoV‐2 (obtained from the World Reference Center for Emerging Viruses and Arboviruses (WRCEVA) at University of Texas Medical Branch (UTMB)) at a final concentration of 100× TCID50 dilution in a BSL‐3 laboratory. Final whole sera and small‐EV treatment concentrations achieved were at ∼160x dilution of small‐EV stocks. After mNG‐SARS‐CoV‐2 infections, the cells were maintained at 37°C and 5% CO_2_ humidity for 72 h. Cell monolayers were washed three times with pre‐warmed 1x PBS and live cells were stained using Hoechst 33342 nuclear stain (Invitrogen, #H3570) for 15 min. Plates were imaged on fluorescent plate reader (Tecan, Infinite 200 Pro) scanning at 488 nm (mNeonGreen) and 350 nm (Hoechst 33342). Cells were then fixed O/N at room temperature with 4% paraformaldehyde solution and fluorescent cell images were acquired on a Celigo Imaging Cytometer (Nexcelom).

### 2.11 Data analysis

For sequencing data analysis, raw FASTQ data files obtained from an Illumina HiSeq2500 sequencer were processed using the RNAworld server from the Tuschl Laboratory at the Rockefeller University, including adapter trimming and read alignments and annotation. MiRNA counts were exported to spreadsheets for data analysis. Statistical analyses of miRNA counts were performed using dedicated Bioconductor packages in the R platform, as detailed below. Heat maps were generated from transformed counts using the ‘NMF’ package (aheatmap function). Differential expression was assessed using ‘DESeq2’ and ‘edgeR’. Differential expression models included a batch variable (library) to reduce batch biases. Interactions with sex and age were tested (Mensà et al., 2019). To maximize the discrimination ability of miRNA we computed a score for each sample (‘miRNA score’, (Mazeh et al., 2018)), assembled by summing the standardized levels (z‐values) of all significantly upregulated miRNA, and the negative of the z‐values of all significantly downregulated miRNA. For quantitative PCR (qPCR) we subtracted the threshold cycle (Ct) value of ath‐miR‐159a (exogenous normalization control) from the Ct value of each individual miRNA and calculated the ΔΔCt comparative method values. QPCR differential expression was assessed via *t*‐tests or Mann‐Whitney U tests of ΔΔCt values. For convalescent small‐EV neutralization assays, statistical analysis was performed using Graphpad Prism 8.0 software, where data was assessed using mean ± standard error of the mean (SEM). All in vitro experiments consisted of both technical (three separate wells) and biological (three separate experiments) replicates. One‐way analysis of variance (ANOVA) with Bonferroni post hoc test was conducted. A *P*‐value < 0.05 was considered statistically significant.

## 3 RESULTS

### 3.1 Development of the EV‐CATCHER assay for purification of small‐EVs from human biofluids

**FIGURE 1:**
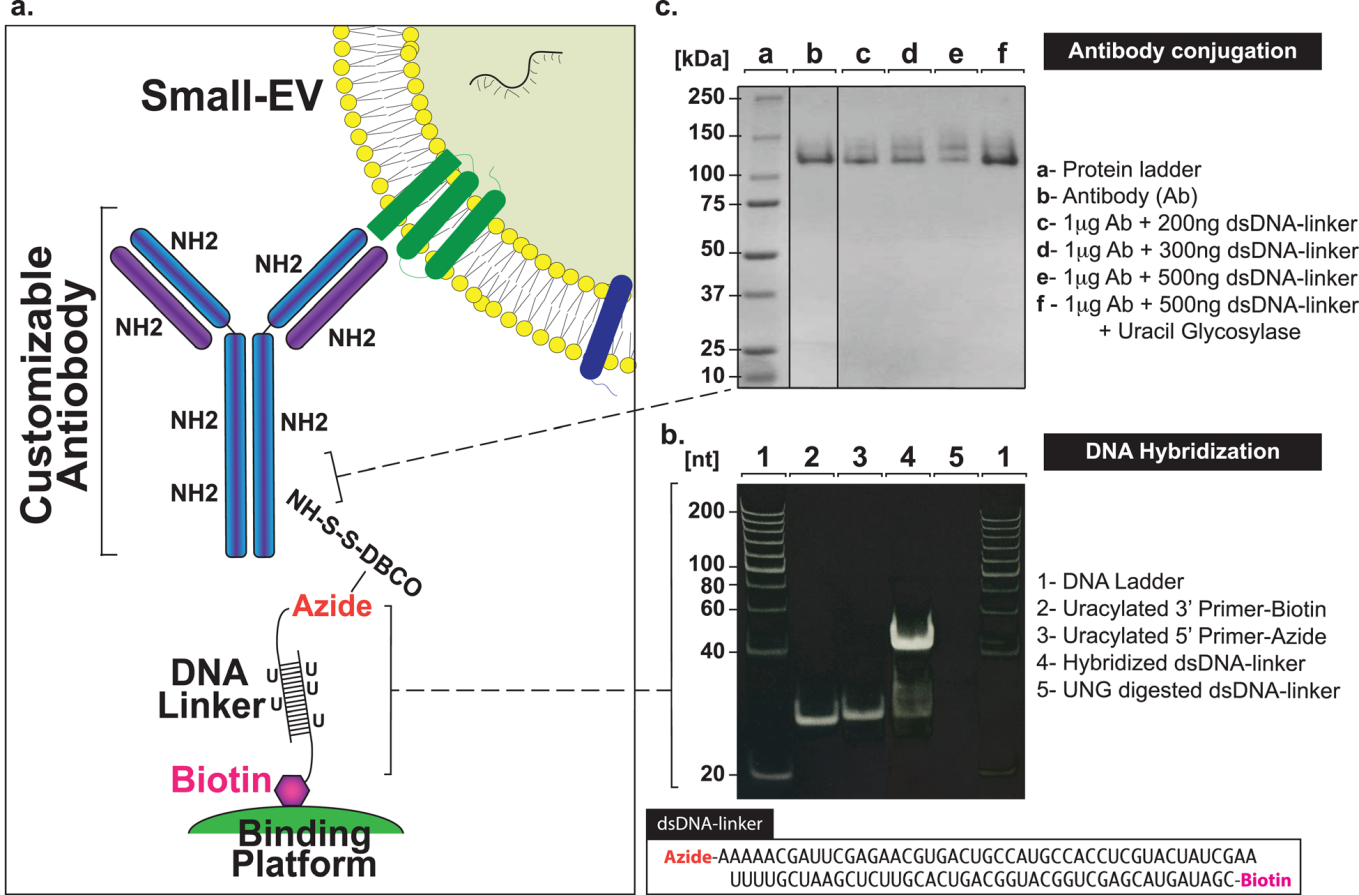
Strategy developed for selectively purifying small‐EVs from human biofluids. a. Schematic representation of the EV‐CATCHER assay designed for purification of small‐EVs from biofluids, which relies on binding of a degradable dsDNA‐linker (uracilated 5′‐azide oligonucleotide annealed to complementary uracilated 3′‐biotin oligonucleotide) to a DBCO‐activated antibody and to a streptavidin‐coated platform. b. Acrylamide gel migration of single stranded oligonucleotides and double stranded (ds) hybridized DNA‐linker. Single stranded (ss) biotin labelled DNA (lane 2); ssDNA azide (lane 3), dsDNA linker (lane 4), and UNG digested dsDNA linker (lane 5) were separated on a 15% non‐denaturing PAGE gel. c. Monoclonal human anti‐CD63 antibody (Ab) activation, for selection of small‐EVs expressing CD63, and dsDNA‐linker conjugation. Representative image of Coomassie stained non‐denaturing PAGE gel (12%) with migration of protein ladder (lane a), 1 μg native anti‐CD63 Ab (lane b); dsDNA‐linker conjugated to 1 μg DBCO‐activated anti‐CD63 antibody using increased amounts of dsDNA‐linker (lanes c‐e), dsDNA‐linker‐Ab (500 ng ‐ 1 μg) digested by UNG (lane f). Experiments were replicated and imaged on a ChemiDoc MP imager

We endeavoured to optimize the customizable attachment of selective antibodies to a binding platform for specific purification of small‐EVs from biological fluids. Thus, using the design described by Löf et al., 2017 (Figure 1a) we initially established optimal hybridizing conditions to generate the highest amount of the double‐stranded DNA linker for subsequent assays (Figure 1b, lane 4) (Löf et al., 2017). In order to increase purity of the dsDNA and to prevent representation of single‐stranded 5′ azide oligonucleotides, which could bind to the DBCO‐activated antibodies and prevent their binding to the platform, we purified the annealed dsDNA products on a non‐denaturing acrylamide gel. Using uracil‐glycosylase (UNG), we validated the degradable nature of the dsDNA linker through enzymatic digestion (Figure 1b, lane 5). Next, we determined the optimal ratio between dsDNA‐linker and the DBCO activated antibodies, which was evaluated on a 12% PAGE (Figure 1c, compare lanes c, d and e). The binding of the ds‐DNA linker modified the migratory pattern of the antibody, which could be restored upon UNG glycosylase treatment and digestion of the dsDNA linker (Figure 1c, lanes f). These experiments were repeated with different antibodies and validated our optimized conditions for preparation of ds‐DNA linker and antibody duplexes.

### 3.2 Identification of a low non‐specific nucleic acid and small‐EV binding platform

Although magnetic beads are extensively used for the isolation of small‐EVs and exosomes prior to the evaluation of their cargos, repeated experiments conducted in our laboratory suggested high‐levels of non‐specific small‐RNA binding in the absence of a capture antibody (Supplementary Figure 1). Thus, we sought to investigate the small‐RNA and small‐EV non‐specific binding capacity of magnetic beads from several commercially available kits by comparison to the non‐magnetic streptavidin coated 96‐well plates used with EV‐CATCHER (wells; largely used for ELISA assays), as described in Figure 2a. We chose commercial kits widely‐used in peer‐reviewed publications, which included magnetic‐beads of sizes ranging from 130 nm to 9μm, but also included global purification methods and kits (Figure 2a., ExoEasy, ExoQuick, Ultracentrifugation) as controls. For our first evaluations on the non‐specific binding of small‐RNAs to magnetic beads we used a synthetic ath‐miR‐159a RNA oligonucleotide (100pg/assay), an *Arabidopsis‐thaliana* miRNA that is not found as a contaminant in the laboratory (control PCR experiments). We selected the four kits providing customizable magnetic‐beads (the manufacturer sells them without conjugated antibodies) including Dynabeads MyOne Streptavidin and carboxylic acid T1 beads, MojoSort beads, and ExoCap beads, in comparison to Streptavidin‐coated 96 well‐plates (EV‐CATCHER), as displayed in Figure 2b. Using qPCR as a sensitive measuring tool, we detected ath‐miR‐159a (fold change by comparison with control (total amount added)), for all methods following 3 washes and the final elution from the magnetic beads/wells. We tested the magnetic‐beads and wells as purchased without antibodies (Figure 2b. left), in presence of BSA (Figure 2b. centre), and in presence of BSA+ RNase‐A (Figure 2b. right) and evaluated the non‐specific binding of ath‐miR‐159a by RT‐qPCR. Although the addition of BSA (1%) or BSA (1%) + RNase‐A decreased non‐specific binding of the ath‐miR‐159a (Figure 2b. middle and right graphs), the Streptavidin‐coated 96 well‐plates used with EV‐CATCHER displayed the lowest non‐specific binding by more than 10 PCR cycles. Next, we sought to evaluate the non‐specific binding of 100pg ath‐miR‐159a spiked into 100μl of human serum, but this time by using all 11 selected methods, in their small‐EV purification configuration with magnetic‐beads conjugated to antibodies (anti‐Tim4 antibody for MagCapture, and anti‐CD63 antibody for customizable and customized methods, as displayed in Figure 2a.), by comparison to our anti‐CD63 EV‐CATCHER assay (Figure 2c). We also included global small‐EV purification methods such as ExoEasy, Nörgen Exosome kit, ExoQuick, and ultracentrifugation, as comparative controls. RT‐qPCR data of ath‐miR‐159a, spiked into serum by comparison to RNase‐free 1×PBS, suggests RNA degradation as Comparative Threshold detection (Ct) values increased from 10 to 22 in our control (See Figure 2b, First black bar). However, globally, all methods and especially all magnetic‐beads selected for these analyses, demonstrated non‐specific purification of ath‐miR‐159a as Ct values ranged between 26–34, with the highest Ct values for ultracentrifugation (Ct = 35) and CD63+ EV‐CATCHER with a Ct of 38. These experiments further highlight the non‐specific capacity of magnetic‐beads to bind small‐RNAs, even when conjugated to their small‐EV purification antibody. Considering that small‐RNAs, protected from degradation in serum, can be found in small‐EVs, we then sought to determine if magnetic‐beads could also non‐specifically bind small‐EVs, and further increase small‐RNA non‐specific signal (Figure 2d.). For these experiments we used the same four different commercial magnetic‐beads as described in Figure 2b., since all other magnetic bead‐based methods came pre‐manufactured with capture antibodies targeting small‐EV surface markers (anti‐Tim4 antibody for MagCapture and antiCD63 antibody for other methods; See Figure 2a.), which would interfere with our measures. We chose to perform RT‐qPCR detection of hsa‐miR‐21 and hsa‐miR‐200c, which we previously measured by small‐RNA sequencing in RNA extracts of small‐EVs from MCF‐7 cells. The schematic displayed in Figure 2d describes how we measured these two miRNAs: 1‐ for non‐specific binding to the platforms (wells (EV‐CATCHER) or magnetic‐beads) by elution after 3 washes, and 2‐ by measuring the amounts of these miRNAs remaining in solution after non‐specific capture. Considering that commercially purified MCF‐7 small‐EVs likely underwent damage during processing, we expected free hsa‐miR‐21 and hsa‐miR‐200c in the small‐EV aliquoted solutions and thus pre‐treated all reactions with RNase‐A, so to only measure these small‐EV bound hsa‐miR‐21 and hsa‐miR‐200c (See Figure 2d compare grey and black bars in all graphs). For non‐specific capture of small‐EVs (Figure 2d left graphs top and bottom), we observed that EV‐CATCHER and ExoCap provided the lowest detection of hsa‐miR‐21 and hsa‐miR‐200c and thus non‐specific capture of small‐EVs, by comparison to the three other magnetic‐bead purifications. Comparatively, when measuring the amounts of hsa‐miR‐21 and hsa‐miR‐200c remaining from the initial small‐EV solutions, we determined that EV‐CATCHER had the most amount of both miRNAs (See Figure 2d right graphs top and bottom), and confirmed the low‐binding properties of the streptavidin coated 96 well‐plates used with our method. It is important to note that a portion of non‐specifically bound small‐EVs are removed during the three standard 1xPBS washes from the magnetic beads, and account for quantifiable losses that as previously identified by qPCR (see Supplementary Figure 2). We also observed that adding BSA (1%) lowered non‐specific capture of small‐EVs by all magnetic bead‐based methods, but the wells still retained the lowest amounts of small‐EVs as indirectly quantified by qPCR (See Supplementary Figure 3). Additionally, we performed an extensive evaluation of the small‐EVs purified by all 12 different methods (8 methods by immuno‐capture including EV‐CATCHER, 2 column‐based capture, ExoQuick, and Ultracentrifugation) using Western blotting and evaluated small‐EV proteins that included CD63, CD9, and CD81, as well as contaminant proteins that included Albumin, and both lipoproteins ApoA1 and ApoB (Figure 2e.). Small amounts of ApoA1 and Albumin were detectable after EV‐CATCHER purification, however lower than with most commercial methods. We quantified the number and size of the small‐EVs purified from human serum by CD63^+^ EV‐CATCHER and 6 other methods (MagCapture CD63^+^, ExoFlow CD63^+^, ExoEasy, the Nörgen exosome purification kit, ExoQuick, and ultracentrifugation). Our analyses revealed the highest number of particles for ExoQuick and the Nörgen kits as anticipated, with particles of larger size due to bulk small‐EV purification (See Figure 2f). EV‐CATCHER (enzymatic release of small‐EVs) appeared to release more small‐EVs than ExoFlow or MagCapture (See Figure 2f). Finally, using TEM we evaluated the size and morphology of the small‐EVs purified from human serum and released by EV‐CATCHER and 3 other commercial methods including ExoCap, ExoFlow, Dynabeads, and including Ultracentrifugation as a control (Figure 2 g). Altogether, our data show that EV‐CATCHER along with ExoFlow and ExoCap provided high‐quality small‐EVs. However, due to the non‐specific capture of small‐RNAs and small‐EVs by all magnetic bead‐based methods as measured in Figure 2b. and 2c., we selected streptavidin‐coated 96‐well plates for all subsequent experiments for immuno‐purification of small‐EVs, using the EV‐CATCHER assay.

FIGURE 2Identification of a low‐background binding platform and comparison to 11 existing small‐EV purification methods. a. Diagram comparing EV‐CATCHER to 10 commercially available kits and ultracentrifugation for purification of small‐EVs. Three magnetic‐bead purification methods were identified where chemical treatment have been described (1% Formic Acid pH 2.0‐ 4.0, 0.1 M Glycine pH 2.5‐3.0 (Chen et al., 2020; Hisey et al., 2018; Hong et al., 2014), or proprietary buffer (SBI ExoFlow)) for small‐EV release (Barros et al., 2018; Burkova et al., 2019; Gutzeit et al., 2014; Hardin et al., 2018; Huang et al., 2018; Mallegol et al., 2007; Patel et al., 2019; Peterson et al., 2015; Theodoraki et al., 2018; Velandia‐Romero et al., 2020; ). b. Evaluation of non‐specific small‐RNA binding to four commercially different customizable magnetic beads (130 nm to 4.5 μm diameter), including both streptavidin‐coated (3 methods) and Carboxyl‐coated beads (1 method), by comparison to wells used for the EV‐CATCHER assay. A 1xPBS solution containing 100 pg ath‐miR‐159a RNA oligonucleotide was incubated with wells (1‐ EV‐CATCHER in triplicate) or the four different types of customizable magnetic‐beads (2‐ MojoSort, 3‐ Carboxyl‐Dynabeads, 4‐ ExoCap, 5‐ Streptavidin‐coated Dynabeads) in triplicate. Wells and magnetic‐beads were washed 3 times and the eluted RNA was quantified by RT‐qPCR (Left graph). Two additional conditions were tested to prevent non‐specific binding of ath‐miR‐159a including a pre‐treatment with BSA (1%) (middle graph), and a combined pre‐treatment with BSA (1%) and RNase‐A (12.5 μg/ml) (Right graph). c. Evaluation of non‐specific binding of ath‐miR‐159a as a contaminant in 100 μl of human serum for all 10 commercial kits (#2 to #11) and the ultracentrifugation method (#12). All magnetic‐bead kits were tested in their ‘Exosome’ purification commercial forms with antibodies on their surface (anti‐Tim4 for Fujifilm and CD63 for all other techniques) and compared to anti‐CD63 EV‐CATCHER (#1). Following manufacturer's instructions, small‐EVs were eluted and RNA was extracted prior to RT‐qPCR quantification of ath‐miR‐159a. All experiments were repeated three times. The averaged comparative thresholds (Ct) values are added above the standard deviation bars in the graph, for each commercial kit and method. d. Evaluation of non‐specific binding of MCF‐7 small‐EVs to EV‐CATCHER and 4 customizable small‐EV commercial magnetic‐bead purification assays. The experimental collection of non‐specifically bound small‐EVs or small‐EVs remaining in solution after non‐specific capture with EV‐CATCHER and the 4 magnetic bead‐based commercial kits is displayed in the schematic (above). RNase A (12.5 μg/ml) was added prior to all experiments, to remove free‐floating miRNAs (Compare grey bars to black bars in all graphs). The top two graphs represent RT‐qPCR quantifications of hsa‐miR‐21 from wells or magnetic beads exposed to the MCF7 solution containing 10 μg of small‐EVs/ ‘exosomes’(left) or remaining in the solution (right), after RNA extraction (All experiments were performed in triplicate). The lower two graphs represent the same conditions with quantification of hsa‐miR‐200c (Non‐specific binding to wells or magnetic beads (left) or remaining in solution (right). All RT‐qPCR experiments were performed in triplicate for each method. Data is represented as fold change with technical replicates (subtraction of ath‐miR‐159a and 10 μg MCF‐7 control). e. Western blot analyses (8 μg total protein) of small‐EVs purified by CD63 EV‐CATCHER and all commercial kits (Tim4 antibody for Fujifilm, and CD63 antibody for all other magnetic bead‐based methods) and methods (ExoEasy, Nörgen, ExoQuick, ultracentrifugation). Anti‐human ‐ApoB, ‐CD63, ‐Albumin, ‐ApoA, ‐CD9, ‐CD81 antibodies were used to evaluate the small‐EVs purified by each of the 11 selected methods/commercial kits. f. Nanoparticles tracking of released small‐EVs using a Spectradyne NT instrument. Small‐EVs evaluated were obtained by EV‐CATCHER (anti‐CD63), MagCapture (anti‐CD63), ExoFlow (anti‐CD63), and global ExoEasy, Norgen purification kit, ExoQuick, and ultracentrifugation purifications methods. g. Transmission Electron Microscopy (TEM) with direct magnification of 20,000x and scale bars of 100 nm for small‐EVs purified from human serum using EV‐CATCHER (anti‐CD63, duplicated isolations), Exoflow (anti‐CD63), ExoCap (anti‐CD63), Dynabeads T1 MyOne™ beads (anti‐CD63) and ultracentrifugation

**Figure:**
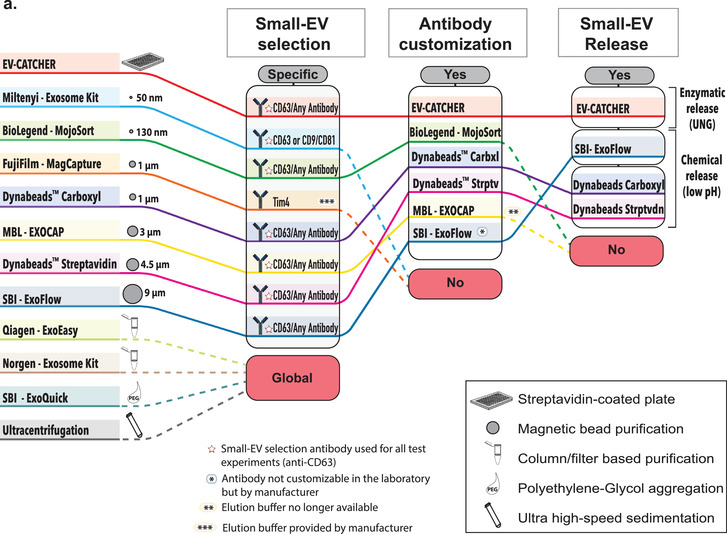

**Figure:**
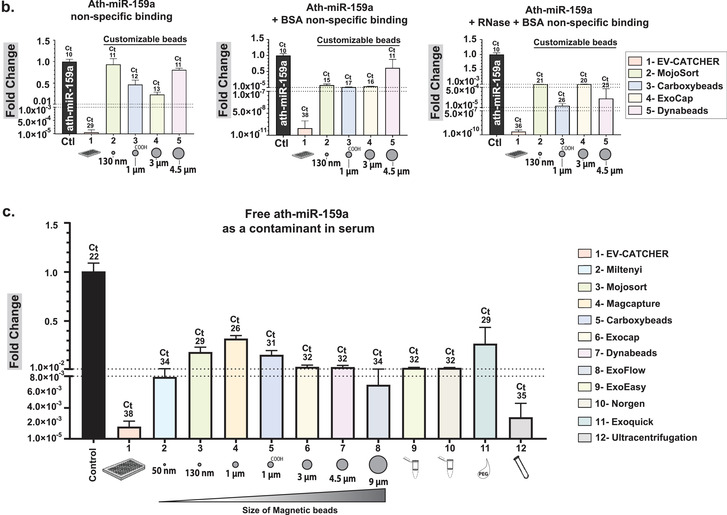

**Figure:**
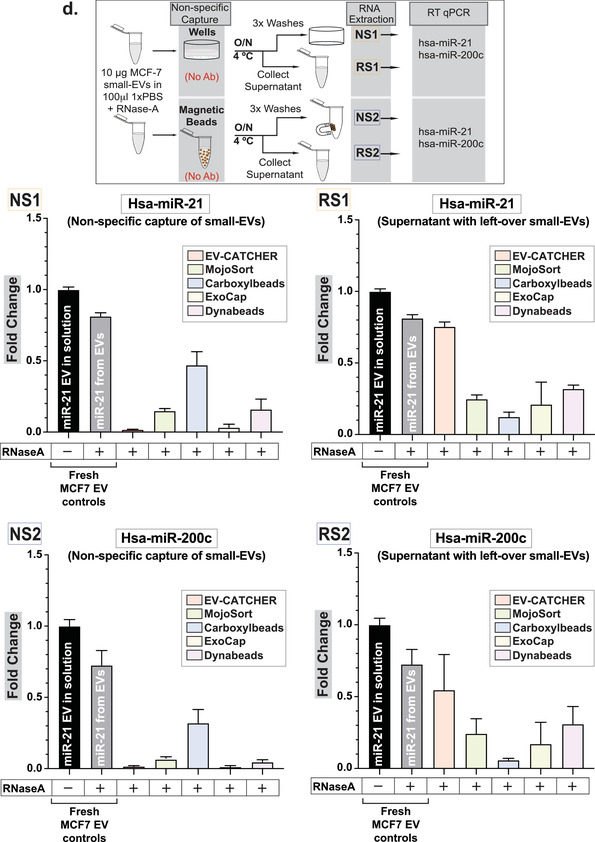

**Figure:**
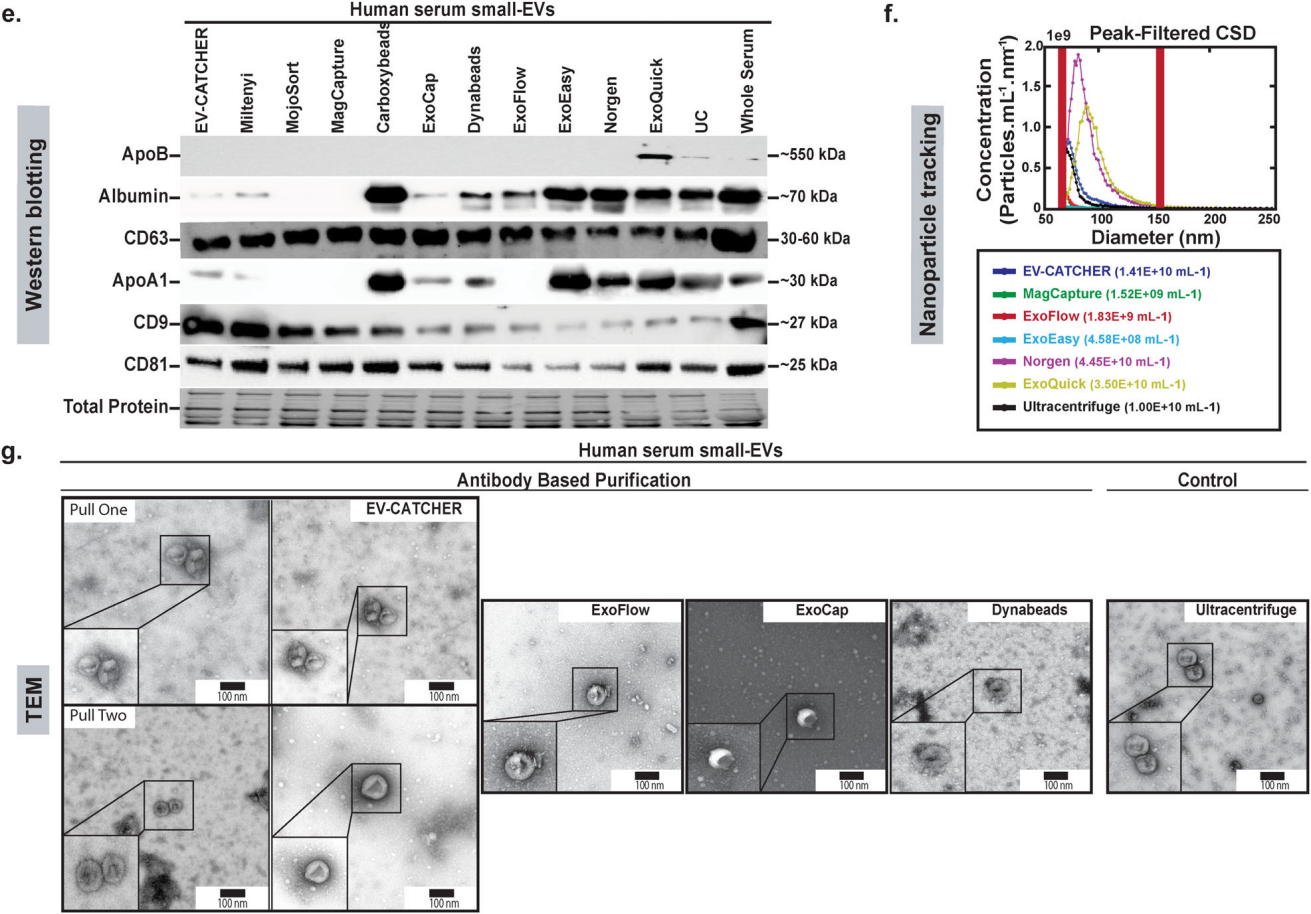

### 3.3 Reproducibility and sensitivity of the EV‐CATCHER assay for purification of small‐EVs from biological fluids

**FIGURE 3:**
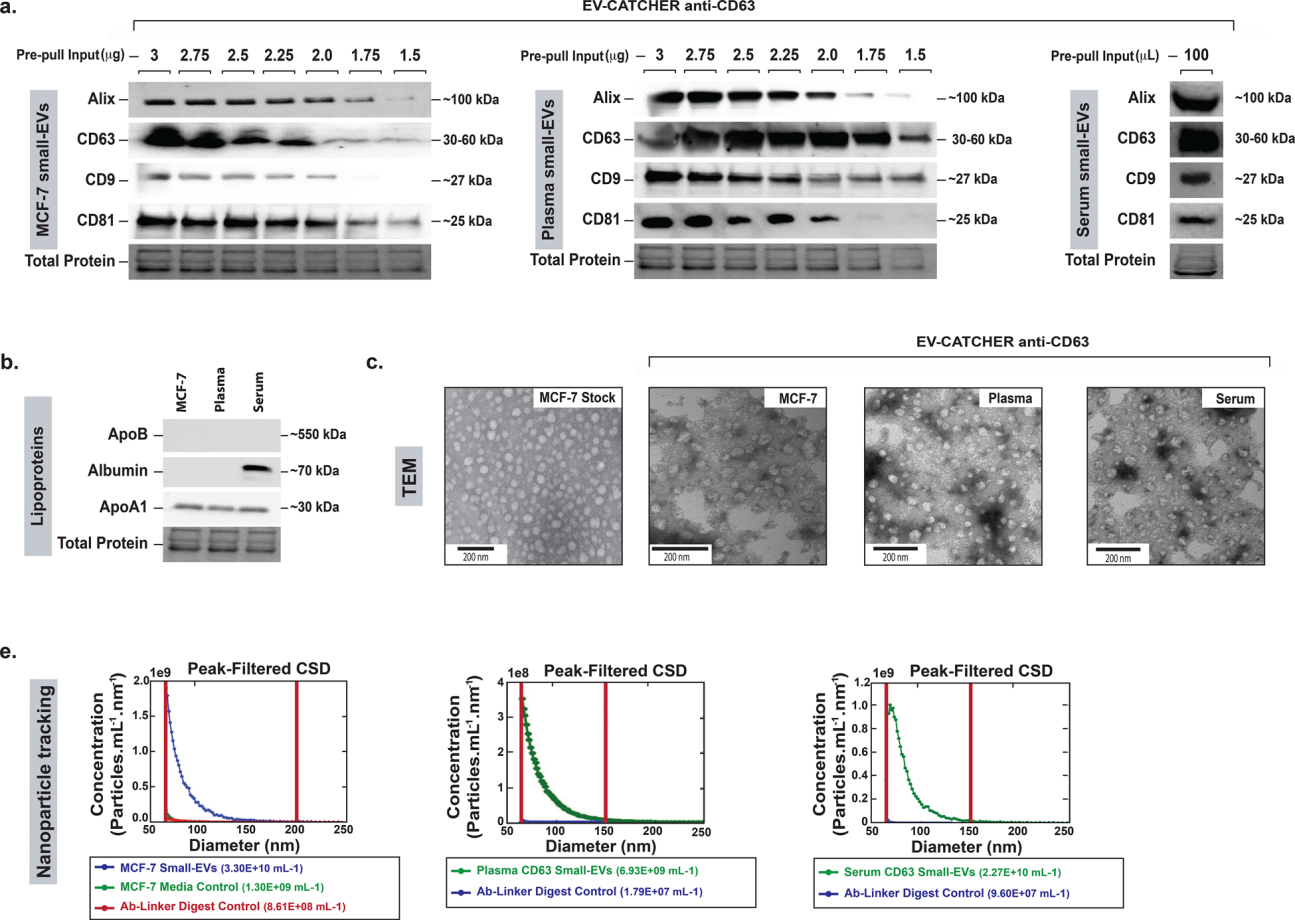
Evaluation of small‐EV purification reproducibility using EV‐CATCHER with different biological fluids. a. Western blot evaluation of small‐EV surface markers using anti‐Alix, ‐CD63, ‐CD9, and ‐CD81 antibodies. EV‐CATCHER purification of CD63^+^ small‐EVs from MCF‐7 tissue culture media (left) and human plasma (middle) with decreasing total protein inputs before purification. For left and middle Western blots, 3 μg (lane 1), 2.75 μg (lane 2), 2.5 μg (lane 3), 2.25 μg (lane 4), 2 μg (lane 5), 1.75 μg (lane 6), and 1.5 μg (lane 7) total protein was used before EV‐CATCHER purification. EV‐CATCHER was also used for purification of CD63^+^ small‐EVs from a serum sample, and validated by the four different surface protein antibodies b. Western blot evaluation of ApoB, Albumin, and ApoA1 proteins from MCF7, human plasma, and human serum CD63^+^ small‐EVs purified with EV‐CATCHER. c. Transmission electron microscopy (TEM) of MCF‐7 small‐EV stock (left, MCF‐7 Stock) and anti‐CD63 EV‐CATCHER purified CD63^+^ small‐EVs from MCF‐7 small‐EV stock (MCF‐7), CD63^+^ small‐EVs from human plasma (Plasma), and CD63^+^ small‐EVs from human serum (Serum). Direct magnification of 20,000x and scale bars of 200 nm are represented on the TEM images. c. Representative particle size distribution characterized by nanoparticle tracking (Spectradyne nCS1 equipped with TS400 microfluidic cartridges) for anti‐CD63 EV‐CATCHER‐purified small‐EVs from MCF‐7 small‐EV stock (left graph), human plasma (middle graph), and human serum (right graph). Peak filtering performed for diameter < 65 nm and transit times > 80 μs. The concentrations are representative of particles detected between 65 and 150 nm

For these proof‐of‐principle experiments, we used an anti‐CD63 antibody for purifications performed with the EV‐CATCHER assay and thus evaluated CD63^+^ small‐EVs from three different biological sources (tissue culture media of a human cell line (MCF‐7), human plasma, and human serum) (Figure 3). We performed these purifications in the presence of decreasing amounts of total protein inputs (quantified by Nanodrop 2000 spectrophotometer) and evaluated the reproducibility of the CD63 purifications by Western blotting, using anti‐Alix, ‐CD63, ‐CD9 and ‐CD81 antibodies for protein expression in the small‐EV elutions (Figure 3a). Small‐EV purification from serum was performed with a single volume of 100 μl, due to limited availability. We also evaluated the presence of protein contaminants including ApoB, Albumin, and ApoA1 by Western blotting of small‐EVs purified from MCF‐7 small‐EVs (SBI), human plasma, and human serum (Figure 3b). We then used transmission electron microscopy (TEM, Figure 3c) to reveal the presence of intact small‐EVs with size and morphology consistent between the different purifications (Williams et al., 2013). Finally, using a Spectradyne nCS1 nanoparticle tracker we evaluated the size distribution of the CD63^+^ purified and released small‐EVs, which revealed the presence of nanoparticles with sizes ranging between 65–150 nm for small‐EVs from MCF‐7 cell media (3.30 × 10^10^ particles/ml), human plasma (6.93 × 10^9^ particles/ml, and human serum (2.27 × 10^10^ particles/ml) by comparison to UNG digested Ab‐DNA linker from the wells incubated with 1x PBS (Figure 3d). Collectively these analyses revealed uniform reproducibility of the EV‐CATCHER assay for purifying small‐EVs from different biofluids using an anti‐CD63 antibody, with specificity provided by the selection antibody (Supplementary Figure 4).

### 3.4 Small‐RNA sequencing using RNA extracted from CD63^+^ small‐EVs purified with EV‐CATCHER

**FIGURE 4:**
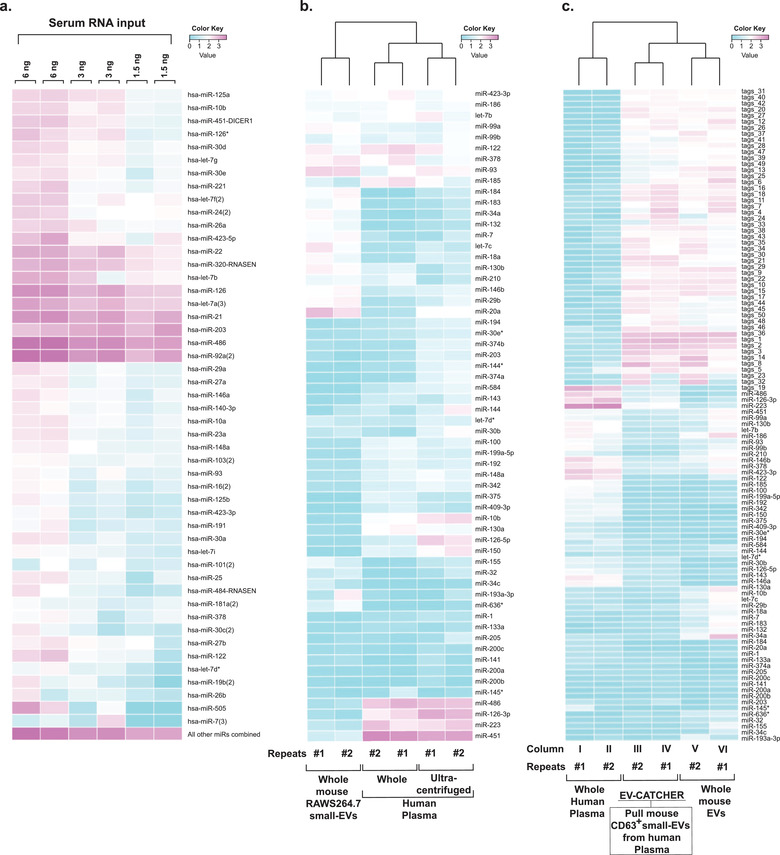
Small‐RNA sequencing of small‐EVs purified from biofluids. a. Next‐generation sequencing and heatmap representation of the top expressed circulating miRNAs in human serum. The heat map includes the top 49 expressed miRNAs and their detectability using decreasing amounts of total RNA extracted from human serum (6 ng, 3 ng, and 1.5 ng, in duplicate). b. Heat map representation displaying miRNA expression differences between total RNA extracted from mouse RAWS264.7 tissue culture EVs (first two rows), commercially available whole human plasma (third and fourth rows; same plasma as the one analysed in Figure 3), and small EVs obtained by ultracentrifugation from the same commercially available whole human plasma sample (fifth and sixth rows). Duplicate libraries were obtained using newer (Repeats#1) and older (Repeats#2) 3′ barcoded adapters. c. EV‐CATCHER specific capture of mouse RAWS264.7 CD63^+^ small‐EVs, which represent a subset of all CD63^+^ small‐EVs, from the RAWS264.7 total small‐EVs that were spiked into human plasma (same plasma as the one evaluated in Figure 3). The heatmap represents miRNA expression differences between total RNA from human plasma (columns I and II) and total RNA from mouse RAWS264.7 total small‐EVs (columns V and VI) and include the top 58 human plasma‐specific miRNAs and the top 50 mouse RAWS264.7 small‐EV specific transcripts differentially expressed between total RNA from human plasma and total RNA from mouse RAWS264.7 total small‐EVs, respectively. Small‐RNA sequencing profile of CD63^+^ mouse RAWS264.7 small‐EVs (1 μg) purified from human plasma (100 μl) using the CD63^+^ EV‐CATCHER assay harbouring a mouse‐specific anti‐CD63 antibody (columns III and IV). Duplicate libraries were obtained using newer (Repeats#1) and older (Repeats#2) 3′ barcoded adapters. Data was analysed using dedicated Bioconductor packages in the R platform and heatmaps were generated using the ‘NMF’ package (a heatmap function)

Considering that our previous small‐RNA sequencing experiments using total RNA extracted from small‐EVs purified from serum with unconjugated magnetic beads (Supplementary Figure 1) indicated the presence of high small‐RNA background, we sought to evaluate the specificity of the EV‐CATCHER assay using the same small‐RNA cDNA library preparation protocol (Loudig et al., 2017; Loudig et al., 2018). As an initial test we validated the applicability of our cDNA library preparation protocol to the detection of circulating small‐RNAs using decreasing amounts of total RNA extracted from whole serum (Figure 4a). Duplicate experiments demonstrated the reproducibility of our sequencing pipeline with small‐amounts of circulating small‐RNA. Next, we compared small‐RNA sequencing data of total RNA extracted from whole plasma (same human plasma as the one detailed in Figure 3) or from small‐EVs ultracentrifuged from human plasma (Supplementary Figure 5) and identified specific miRNA expression differences (Figure 4b), which further corroborated that the RNA extraction method influences the expression output (El‐Khoury et al., 2016). As a measure of control, we validated detectability of specific small‐RNA expression differences between mouse cell culture and human plasma small‐EVs (Figure 4b). Finally, we sought to determine if we could specifically select a sub‐population of small‐EVs from a biofluid using EV‐CATCHER. For these experiments we chose to spike human plasma with commercial mouse RAWS264.7 small‐EVs (SBI). We customized the EV‐CATCHER assay with an anti‐mouse CD63 antibody, without cross‐reactivity to human CD63, and carried out the purification of CD63+ mouse small‐EVs from human plasma. For sensitive evaluation of the purified miRNA cargos, we used our cDNA library preparation protocol and sequenced the small‐RNA content of the selected small‐EVs (Figure 4c). For these experiments, all libraries were duplicated using fresh (Repeats #1, 3 years old) and older (Repeats #2, 6 years old) 3′ barcoded adapters to accentuate inherent variabilities of the cDNA library preparation protocol. As observed in Figure 4c, small‐RNA expression data from mouse CD63^+^ small‐EVs (columns III and IV) captured from human plasma using the EV‐CATCHER assay contained mouse‐specific small‐RNA sequences (Tags), identified solely in mouse RAWS264.7 small‐EVs (columns V and VI). We also noted that miRNAs differentially detected in total RNA from whole human plasma (Figure 4c, columns I and II) were not detected within our mouse CD63^+^ small‐EVs purified by EV‐CATCHER (Figure 4c, columns III and IV). Interestingly, some miRNA expression differences were noted between total RNA from RAWS264.7 small‐EVs (representing a pool of all small‐EVs present in the sample; Figure 4c, columns V and VI) and total RNA extracted from CD63^+^ small‐EVs (representing a subset of all small‐EVs; Figure 4c, columns III and IV), which represented specific miRNAs from non‐captured CD63^–^ mouse RAWS264.7 small‐EVs. These next‐generation sequencing experiments demonstrate the reproducible detection of circulating small‐RNAs and specificity of the EV‐CATCHER assay for antibody‐based selected capture of a CD63^+^ small‐EV subpopulation from biofluids.

### 3.5 MiRNA analysis of small‐EVs purified from the serum of mildly and severely ill Covid‐19 patients

**FIGURE 5:**
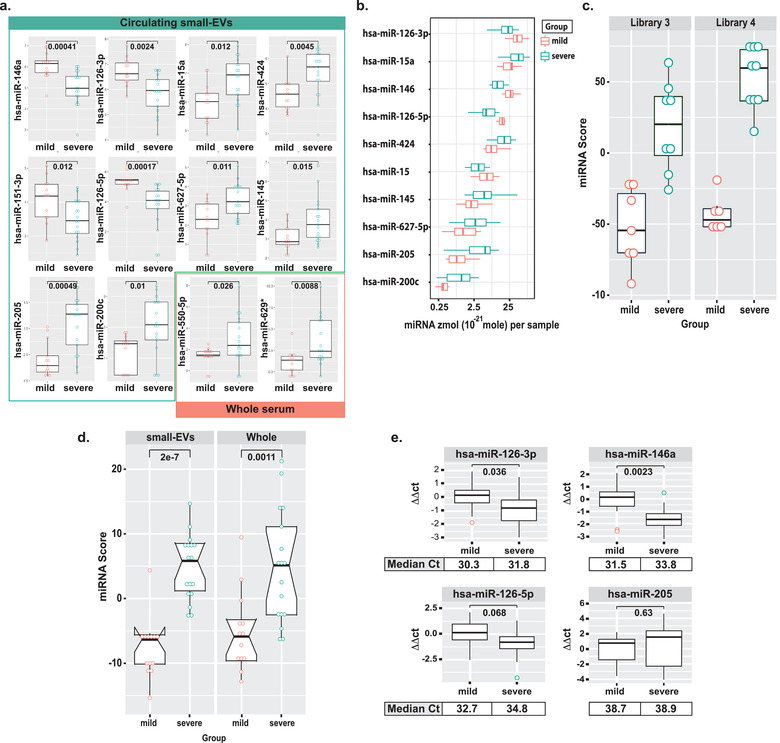
The EV‐CATCHER assay allows for identification of miRNA expression differences between total RNA from small‐EVs and whole serum. a. Individual box plot analyses of the 10 differentially expressed miRNAs identified from total RNA extracted from CD63^+^/CD9^+^/CD81^+^ small‐EVs purified from serum with EV‐CATCHER (green square; hsa‐miR‐146a, hsa‐miR‐126‐3p, hsa‐miR‐424, hsa‐miR‐151‐3p, hsa‐miR‐126‐5p, hsa‐miR‐627‐5p, hsa‐miR‐145, hsa‐miR‐205, and hsa‐miR‐200c) and 2 differentially expressed miRNAs identified from total RNA from whole serum (orange square; hsa‐miR‐550‐5p, hsa‐miR‐629*) of the same mildly and severely ill Covid‐19 hospitalized patients (first set of Covid‐19 serum samples), by next‐generation small‐RNA sequencing. b. Box‐plot of miRNA amount representation (in zeptomoles (10^–21^ mole) of the top 10 differentially expressed miRNAs identified from small‐EVs purified with EV‐CATCHER from the serum of mildly (n = 13) and severely ill Covid‐19 hospitalized patients (n = 17), per sample. c. Box plots representation of the miRNA integrative signature (including the top 10 miRNAs identified in (a.)) between the two small‐RNA libraries prepared with RNA extracted from small‐EVs purified from mild and severely ill patients (library #3 includes 7 mild cases and 8 severe cases, and library #4 includes 6 mild cases and 9 severe cases), displaying significant differences between the two patient groups. d. Box plot representation of the integrative miRNA signature (10 miRNAs) between total RNA extracted from CD63^+^/CD9^+^/CD81^+^ small‐EVs (subset of all CD63^+^/CD9^+^/CD81^+^ small‐EVs in the serum samples) purified with EV‐CATCHER and total RNA extracted from whole sera between mildly and severely ill patients. e. Box plot RT‐qPCR validations of the top 4 differentially expressed miRNAs identified between mild and severely ill Covid‐19 hospitalized patients by small‐RNA sequencing, with RNA extracted from small‐EVs purified with EV‐CATCHER. Data are represented as ΔΔCt with technical triplicates performed for each individual sample. Differential expression was assessed with DESeq2 (R/Bioconductor package) for sequencing results and t‐tests for miRNA score and qPCR results. Median comparative threshold (Ct) for all samples from each group (mildly or severely ill hospitalized patients) is presented in boxes below box‐plots

The location of our institute and its parent hospital network within the early U.S. epicentre of the SARS‐CoV‐2 pandemic, afforded us with the unique opportunity to explore pressing biological questions and further investigate the potential clinical utility of the EV‐CATCHER assay for identification of miRNA biomarkers in serum samples from Covid‐19 patients admitted within our network. An important question with Covid‐19 infection (which has been associated with a wide range of patient outcomes) was to evaluate if circulating miRNA expression changes could be useful for early identification of patients at risk of severe disease. As such we established our first pilot study where we purified circulating CD63^+^/CD81^+^/CD9^+^ small‐EVs, a subset of small‐EVs positive for these tetraspanins and of all small‐EVs, from the serum of mildly and severely affected hospitalized Covid‐19 patients (serum collection at time of hospitalization) and evaluated their miRNA expression content by next‐generation sequencing. For this proof‐of‐principle study, we chose to use a combination of anti‐CD63, ‐CD81, ‐CD9 antibodies for capture of small‐EVs from whole serum. To measure the sensitivity of the EV‐CATCHER assay we measured miRNA expression differences between mildly and severely ill patients using total RNA extracted from circulating small‐EVs (EV‐CATCHER) by comparison to total RNA from whole serum. Mildly ill patients (n = 13) were hospitalized but did not require mechanical ventilation while severely ill patients (n = 17) displayed Acute Respiratory Distress Syndrome (ARDS; following Berlin classification standards (El‐Khoury et al., 2016)) and required mechanical ventilation, and both groups had PCR‐confirmed infection with the SARS‐CoV‐2 virus. Clinical data was obtained from these patients (Supplementary Table 1). Small‐RNA cDNA libraries were prepared with 15 individual samples from RNA extracted from serum small‐EVs (30 samples; 2 libraries) or from whole serum (30 samples; 2 libraries) with each library having even representation of mild and severe RNA samples, to minimize batch effects. Our expression analyses identified 10 differentially expressed miRNAs (p < 0.05) between mild and severe sample groups using total RNA from CD63^+^/CD81^+^/CD9^+^ small‐EVs purified from serum with EV‐CATCHER (Figure 5a; hsa‐miR‐146a (p‐val = 0.00041), hsa‐miR‐126‐3p (p‐val = 0.0024), hsa‐miR‐424 (p‐val = 0.00454), hsa‐miR‐151‐3p (p‐val = 0.012), hsa‐miR‐126‐5p (p‐val = 0.00017), hsa‐miR‐627‐5p (p‐val = 0.011), hsa‐miR‐145 (p‐val = 0.015), hsa‐miR‐205 (0.00049), and hsa‐miR‐200c (p‐val = 0.01)) whereas only two differentially expressed miRNAs (p < 0.05) between mild and severe sample groups were identified when using total RNA from whole serum (Figure 5a; hsa‐miR‐550‐5p (p‐val = 0.026), hsa‐miR‐629* (p‐val = 0.0088)). As shown in Figure 5b, the 10 differentially expressed miRNAs from CD63^+^/CD81^+^/CD9^+^ small‐EVs purified by EV‐CATCHER, between the two groups appeared in low abundance but reproducibly detectable. We established an integrative miRNA signature using the 10 differentially expressed miRNAs detected in serum small‐EVs between mild and severe patients (Figure 5c). Evaluation of this signature between the two different small‐EV small‐RNA libraries (i.e. 15 samples per library) validated expression differences observed between mild and severe sample groups (Figure 5c). Interestingly, when we evaluated this integrative miRNA signature for both CD63^+^/CD81^+^/CD9^+^ serum small‐EVs and whole sera (Figure 5d), we confirmed the significant difference between mild and severe sample groups in serum purified small‐EVs (Figure 5d, p‐val = 2e‐07) but also surprisingly in whole sera (p‐val = 0.011), where these 10 miRNAs individually did not display expression differences between the two patient groups. Finally, we utilized gold‐standard quantitative RT‐qPCR approach to evaluate the top 4 differentially expressed miRNAs identified in serum small‐EVs (Figure 5e). In order to enable full technical validation of our findings, we activated fresh anti‐CD63, ‐CD81 and ‐CD9 antibodies, which had been initially used for purification of serum small‐EVs, and prepared fresh small‐EV purifications using EV‐CATCHER from the same 30 whole sera samples (mild n = 13, severe n = 17) and extracted total RNA again. Using TaqMan^®^ assays we evaluated expression differences of hsa‐miR‐146a, hsa‐miR‐126‐5p, hsa‐miR‐126‐3p, hsa‐miR‐205 and successfully validated the top two differentially expressed miRNAs (hsa‐miR‐146a (pval = 0.0023) and hsa‐miR‐126‐3p (0.036)) (Figure 5e, top two box plots). To evaluate a possible reason for the lack of validation (p‐val > 0.05) for the two other miRNAs (hsa‐miR‐126‐3p and hsa‐miR‐205), we calculated the mean of the Ct values for qPCR reactions on the 30 specimens for all 4 miRNAs. We observed that only the two miRNAs with the lowest Ct values (most expressed) could be validated (Figure 5e top box plots, see Ct values), whereas the two with the highest Ct values could not be validated, which suggested that these miRNA transcripts were below qPCR detection thresholds. Altogether, our sequencing analyses demonstrated that the purified subset of small‐EVs harbouring ‐CD63, ‐CD81 and ‐CD9 tetraspanins, purified from serum, contain low‐abundance specific miRNA transcripts that can be captured using the EV‐CATCHER assay and be validated by qPCR for the most abundant transcripts. Our analyses also suggest a differential miRNA expression between CD63^+^/CD81^+^/CD9^+^ serum small‐EVs from mildly and severely ill Covid‐19 hospitalized patients, which will require further evaluation.

### 3.6 Small‐EVs purified from convalescent sera with EV‐CATCHER maintain neutralizing properties for in vitro analyses

**FIGURE 6:**
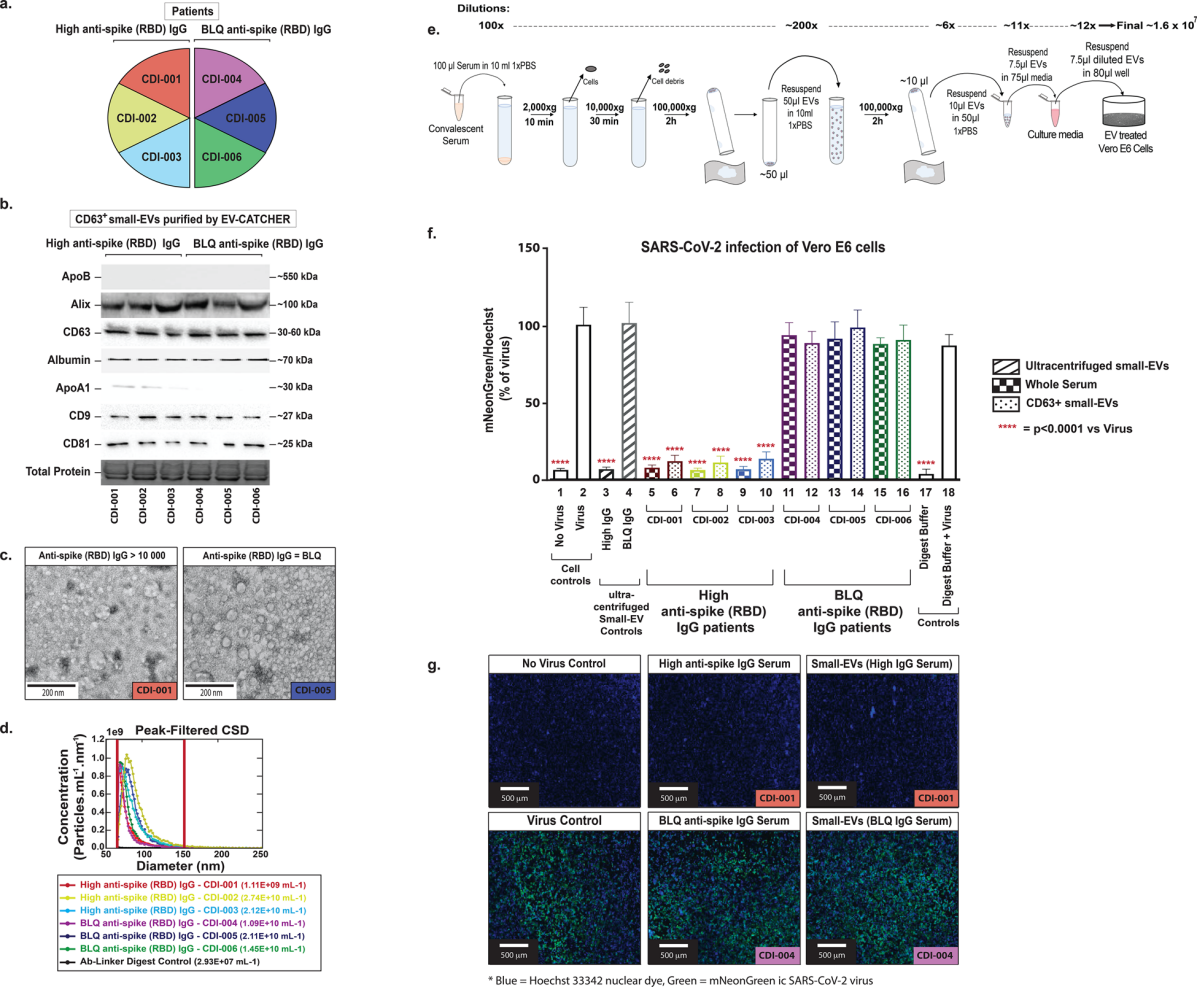
The EV‐CATCHER assay releases functional small‐EVs. a. Six convalescent serum samples (Second set of covid‐19 serum samples) distributed in two groups based on presence of immunoglobulins (IgG) targeting the Receptor Binding Domain (RBD) of SARS‐CoV‐2 spike protein as antigen. Three high anti‐spike IgG titer (left) and three below level of quantification (BLQ) anti‐spike IgG titer serum samples were quantified by ELISA (Dr. Perlin's Laboratory). High anti‐spike IgG titers were estimated with activity remaining at a 10,000x (fold) dilution, while below level of quantification (BLQ) IgG serum samples did not contain detectable IgG against the spike protein RBA region of SARS‐CoV‐2 at the same dilution. b. Western blot analyses of CD63^+^ small‐EVs purified with anti‐CD63 EV‐CATCHER from sera of high anti‐spike (RBD region) IgG (lanes 1–3) and BLQ IgG (lanes 4–6) serum samples using anti‐ApoB, ‐Alix, ‐CD63, ‐Albumin, ‐ApoA1, ‐CD9, and ‐CD81 antibodies. c. Representative Transmission electron microscopy (TEM) images of small‐EVs isolated from high anti‐spike IgG (CDI ‐001, Figure 6b ‐ Lane 1) and BLQ anti‐spike IgG (CDI‐004, Figure 6b ‐ Lane 6) convalescent sera using the anti‐CD63 EV‐CATCHER assay. Direct magnification was 20,000x and scale bars are for 200 nm. d. Nanoparticle size distribution characterized using the Spectradyne nCS1 instrument with a TS400 microfluidic cartridges of CD63^+^ small‐EVs purified from all six serum donors using the anti‐CD63 EV‐CATCHER assay. e. Schematic representation of the experimental procedure used to obtain high‐purity small‐EVs from convalescent sera by ultracentrifugation. f. In vitro assessment of SARS‐CoV‐2 infection using Vero E6 cells treated with whole sera or small‐EVs purified from the different sera. Healthy Vero E6 cells Control (bar 1, no virus) were subjected to mNG SARS‐CoV‐2 (bar 2, virus), small‐EVs ultracentrifuged (EV control) from serum with high anti‐spike IgG (bar 3) and serum with BLQ anti‐spike IgG (bar 4), mNG SARS‐CoV‐2 with whole sera with high anti‐spike IgG (bars 5, 7 and 9) or BLQ anti‐spike IgG (bars 11, 13 and 15), mNG SARS‐CoV‐2 after Vero E6 cells were treated with CD63^+^ small‐EVs purified with anti‐CD63 EV‐CATCHER from high anti‐spike IgG sera (bars 6, 8, and 10), or with CD63^+^ small‐EVs purified with anti‐CD63 EV‐CATCHER from BLQ anti‐spike IgG sera (bars 12, 14, and 16), and finally UNG digest buffer (bar 17) and UNG digest buffer with mNG SARS‐CoV‐2 (bar 18) for 72 h. Statistical analyses were performed using one‐way ANOVA with Bonferroni post hoc correction. All results are presented as mean ± SEM (n = 3), and **** = *P* < 0.0001 vs. virus. g. Fluorescent imaging of Vero E6 cells infected with the mNeonGreen SARS‐CoV‐2 reporter virus. Hoechst 33342 and mNeonGreen was visualized using fluorescent Celigo Cell Imaging. Representative fluorescent images display healthy Vero E6 cells (no virus control), infected with mNG SARS‐CoV‐2 and treated with high anti‐spike IgG whole serum (high anti‐spike IgG serum), infected with mNG SARS‐CoV‐2 and treated with CD63^+^ small‐EVs purified with the anti‐CD63 EV‐CATCHER assay from high anti‐spike IgG serum (High anti‐spike IgG patient# CDI‐001), infected with mNG SARS‐CoV‐2 (virus control), infected with mNG SARS‐CoV‐2 with BLQ anti‐spike IgG whole serum (BLQ IgG serum; Patient # CDI‐004), infected with mNG SARS‐CoV‐2 and treated with CD63^+^ small‐EVs purified with the anti‐CD63 EV‐CATCHER assay from BLQ anti‐spike IgG serum (small‐EVs (BLQ serum)). Scale bars on images was at 500 μM

Considering that our previous TEM analyses suggested that a large proportion of the small‐EVs released after EV‐CATCHER purification were intact, we sought to determine if small‐EVs purified from biofluids maintained their biological properties for in vitro analyses. For these proof‐of‐concept experiments, we established a second pilot study (SARS‐CoV‐2 convalescent serum sample set, second Covid‐19 sample set), where we purified CD63^+^ circulating small‐EVs from sera of six individuals who had recently and fully recovered from SARS‐CoV‐2 infection (enrolled in the convalescent plasma study at HUMC and evaluated for anti‐SARS‐CoV‐2 by ELISA, targeting the Receptor Binding Domain (RBD) of SARS‐CoV‐2 spike protein as antigen (IgG)). Three of the serum samples were obtained from individuals with high anti‐spike IgG titers (High; as defined by positive signal detectable at > 10,000 fold dilution of sera) and 3 serum samples from individuals with anti‐spike IgG levels below limit of quantification (BLQ; not detectable) as shown in Figure 6a. We used our EV‐CATCHER assay to purify CD63^+^ small‐EVs from the different serum samples and evaluated uniform purity between samples by Western blot analyses of ApoB, Alix, Albumin, ApoA1, CD9, CD63 and CD81 proteins (Figure 6b). These experiments revealed homogeneous purification of small‐EVs between high anti‐spike IgG (Figure 6b, lanes 1–3) and BLQ anti‐spike IgG sera (Figure 6b, lanes 4–6). Next, using TEM we assessed the size, morphology, and integrity of the purified small‐EVs (Figure 6c) from both high anti‐spike IgG and BLQ IgG serum samples after enzymatic release. The small‐EV size distribution was also evaluated by nanoparticle tracking (Figure 6d) and revealed similar nanoparticle distributions between 65–150 nm and similar quantities between high anti‐spike IgG (average concentration: 1.99 × 10^10^ particles/ml) and BLQ anti‐spike IgG (1.55 × 10^10^ particles/ml) serum small‐EVs. Then, we performed mNeonGreen SARS‐CoV‐2 reporter virus (viral replication results in production of measurable green fluorescent protein signal) infection and propagation using gold standard Vero E6, African green monkey kidney cells, which harbour high‐levels of the ACE‐2 receptor (Hoffmann et al., 2020), to evaluate the neutralizing properties of the different sera and small‐EVs purified from serum (Figure 6e) (Ng et al., 2003; Xie et al., 2020; Yamate et al., 2005). Treatment of VeroE6 cells with whole convalescent sera from individuals with high anti‐spike IgG titers, prior to SARS‐CoV‐2 infection, resulted in neutralization of the virus (Figure 6e, High IgG), which was not observed when using whole sera from individuals with BLQ IgG titers (Figure 6e, BLQ IgG). In order to evaluate potential neutralizing properties of circulating small‐EVs, we initially obtained highly‐purified small‐EVs by the gold‐standard ultracentrifugation (UC) approach (Supplementary Figure 6) (Burkova et al., 2019). In order to prevent serum IgG carry‐over, we performed successive ultracentrifugation steps and washes, which we estimated resulted in a ∼1.6 × 10^7^‐fold dilution of the initial sera (Figure 6e; 1 high, 1 BLQ). Our experiments revealed that Vero E6 cells pre‐treated with small‐EVs purified by UC from a high anti‐spike IgG titer serum sample lead to a similar neutralization of the SARS‐CoV‐2 virus (Figure 6e, third column) as to that observed with whole sera treatment, whereas cells treated with small‐EVs purified by UC from the BLQ anti‐spike IgG titer serum sample displayed no neutralizing effect on the SARS‐CoV‐2 virus (Figure 6e, fourth column). To confirm that the observed neutralizing effect was solely due to small‐EVs and not carry‐over IgG, we subjected the purified small‐EVs (at different steps of the centrifugation/ultracentrifugation and washes) to our ELISA test for detection of anti‐spike IgG (Supplementary Figure 7) and found that there was no detectable IgG in the final product of the UC experiment. Our data suggested that the neutralization of the SARS‐CoV‐2 virus in vitro was attributable to small‐EVs purified by UC from the high anti‐spike IgG titer sera. Next, we sought to determine if intact small‐EVs purified using our EV‐CATCHER assay could reproduce the neutralization observed with small‐EVs purified by UC from high anti‐spike IgG sera, but not from BLQ anti‐spike IgG sera, in vitro. First, we evaluated that our EV‐CATCHER assay similarly to UC provided pure small‐EVs, and subjected small‐EVs purified from high‐ and low‐ anti‐spike IgG sera to our ELISA assay. We determined that no detectable anti‐spike IgG was purified with the small‐EVs (Supplementary Figure 7). Our analyses of Vero E6 cells treated with small‐EVs purified with anti‐CD63 EV‐CATCHER from high anti‐spike IgG titer sera (Figure 6f. samples 1–3) showed a similar neutralizing effect against SARS‐CoV‐2 in vitro, as to that observed with small‐EVs purified by UC from high anti‐spike IgG titer serum but not from BLQ anti‐spike IgG serum (Figure 6f. samples 4–6). These neutralizing observations were further validated by the differential detection of fluorescent viral particles by imaging (Figure 6 g). Although these unexpected data are suggestive of neutralizing properties for circulating small‐EVs purified from convalescent sera with high anti‐spike IgG titers, the mechanism of action remains to be identified. As further inquiry on small‐EV viral neutralization, we also evaluated small‐EVs purified from mildly and severely ill patients (from our first cohort of Covid‐19 serum samples obtained at an early stage of the infection by comparison to convalescent serum for the second Covid‐19 serum sample cohort). Our analyses of CD63^+^ small‐EVs purified with anti‐CD63 EV‐CATCHER from 6 mildly ill and 6 severely ill patients indicated no neutralization of the SARS‐CoV‐2 virus in‐vitro (Supplementary Figure 8), suggesting that small‐EVs with viral neutralizing properties may be acquired at a later stage of the immune response, as observed when using convalescent serum but requiring further investigation. However, our analyses demonstrate that circulating small‐EVs purified from Covid‐19 convalescent sera and enzymatically released by the EV‐CATCHER assay maintain their neutralizing property comparably to that observed with small‐EVs obtained by ultracentrifugation from one of the convalescent serum samples.

## 4 DISCUSSION

In this study we developed the EV‐CATCHER assay, a sensitive, customizable antibody‐based extracellular vesicle purification procedure. We applied it to the high‐throughput identification of small‐RNA cargos from small‐EVs of a first set of serum samples, and evaluated the intact and functional release of purified small‐EVs from a second set of serum samples.

A significant issue for identification of circulating miRNA biomarkers is the inherent high‐level of surrounding miRNA noise, which results in the “dampening” of their signal in biofluids, especially for the detection of miRNA expression changes by next‐generation sequencing from a population of cells within a large organism (Williams et al., 2013). The evaluation of circulating small‐EVs provides a unique opportunity to evaluate compartmentalized miRNA cargos released by specific cells, however, current laboratory‐based technologies have major caveats (Larssen et al., 2017; Löf et al., 2017; Ludwig et al., 2018; Macías et al., 2019; Patel et al., 2019; Pugholm et al., 2015; Yu et al., 2018). To address these limitations, we optimized the EV‐CATCHER assay with the intent of minimizing RNA background/noise during small‐EV purification and identified a streptavidin‐coated 96‐well plate as our binding platform, as we determined by extensive comparisons with commercially available purification kits where our analyses revealed that magnetic beads capture small‐RNAs and small‐EVs non‐specifically and in turn interfere with downstream molecular analyses. Furthermore, using our optimized small‐RNA cDNA library preparation protocol, we demonstrated the specificity of EV‐CATCHER, for purifying species‐specific small‐EVs, by sequencing total RNA extracted from mouse CD63^+^ small‐EVs selectively purified from human plasma that was initially spiked with mouse RAWS264.7 total small‐EVs. These data strongly suggest a significant potential for use of the EV‐CATCHER assay by selection of small‐EV subpopulations and identification of circulating small‐RNA biomarkers.

Thus, we proposed that a focused selection of encapsulated small‐RNA biomarkers circulating in small‐EVs may allow for fine‐tuned detection of low‐abundance RNA transcripts, due to background noise reduction. Considering that our research institute and its parent institution were located within the early U.S. epicentre of the SARS‐CoV‐2 pandemic, and that no molecular studies evaluated predictive biomarkers for risk of severe disease from Covid‐19 serum specimens, we sought to apply EV‐CATCHER, in a proof‐of‐principle study, to a first set of serum samples obtained from Covid‐19 hospitalized patients with active infections. Our data analyses of small‐EVs purified from the serum of newly infected and hospitalized Covid‐19 patients strongly support the principle that circulating small‐EVs may provide access to encapsulated low‐abundance miRNA biomarkers not significantly detectable from total RNA obtained from whole serum. Indeed, small‐RNA sequencing analyses performed on a circulating subset of CD63^+^/CD81^+^/CD9^+^ small‐EVs, purified by EV‐CATCHER, allowed identification of 10 differentially expressed miRNAs between mildly and severely ill Covid‐19 hospitalized patients, which were not detectable as significantly differentially expressed with small‐RNA from whole sera (first Covid‐19 serum sample set). Interestingly, when we evaluated these 10 miRNAs as an integrative miRNA signature using whole sera miRNA expression sequencing data, we then found that we could discriminate the two patient groups (integrated p‐val > 0.0011). These observations reinforce the idea that inherent miRNA expression differences detectable from encapsulated small‐EVs are dampened when evaluated within a whole‐biofluid, and further demonstrate the need for focused evaluation of specifically purified small‐EVs for discovery of low‐expressed potentially robust and clinically relevant miRNA biomarkers. Considering that qPCR remains one of the gold‐standards for detection of RNA transcripts in clinical assays, it is important to note that it had significant limitations when working with total RNA extracted from circulating small‐EVs purified from small serum sample volumes (100 μl). For example, during qPCR validations of our findings using this first Covid‐19 serum sample set, we observed that the top two differentially expressed miRNAs (hsa‐miR‐146a and hsa‐miR126‐3p) retained statistical significance between the two patient groups (mild vs. severe), whereas hsa‐miR‐205 and hsa‐miR126‐5p lost significance with comparative detection thresholds ∼38‐39 amplification cycles. These evaluations would suggest that the detection of low abundance biomarkers will require; 1‐ the use of more cell‐specific antibodies for the selective enrichment of small‐EV sub‐populations, 2‐ larger biofluid material inputs, and 3‐ a pre‐amplification step to improve qPCR detection thresholds.

Our comparative CD63^+^/CD81^+^/CD9^+^ small‐EV (subset of all small‐EVs) miRNA expression analyses between Covid‐19 mildly affected (n = 13) and severely ill hospitalized patients (n = 17), who required mechanical ventilation (first set of clinical specimens), allowed for identification and qPCR validation of two downregulated miRNAs, hsa‐miR‐146a and hsa‐miR‐126‐3p. Although on a small‐scale these data suggest that downregulated expression of these miRNAs may be associated with severity of the Covid‐19 infection in these patients. Interestingly, previous studies implicated both of these miRNAs as being immune and vascular regulatory miRNAs, associated with inflammation and injurious vascular events ( Alexandru et al., 2020; Arroyo et al., 2020; Benbaibeche et al., 2020; Boldin et al., 2011; Gao et al., 2015; Taganov et al., 2006). For example, studies by Taganov et al. suggest that hsa‐miR‐146a is a molecular brake on inflammation (Taganov et al., 2006). Studies on hsa‐miR‐126 revealed it to be a vascular miRNA, which regulates angiogenesis (Kuhnert et al., 2008) in part via activation of VEGF signalling (Nicoli et al., 2010). More precisely, studies demonstrated that exosomes, a specific population of small‐EVs, secreted by human endothelial progenitor cells were beneficial to lipopolysaccharide‐induced acute lung injury in mice, in part through the delivery of hsa‐miRNA‐126 (both hsa‐miR‐126‐3p and hsa‐miR‐126‐5p) into the injured alveolus (Zhou et al., 2019). Although on a small‐scale, our observations that both hsa‐miR‐146a and hsa‐miR‐126‐3p may be downregulated in severely ill COVID‐19 hospitalized patients on mechanical ventilators, when compared to mildly ill hospitalized patients, are consistent with reported anti‐inflammatory and vascular health‐promoting properties of these regulators. These observations suggest that a larger‐scale evaluation of serum circulating small‐EVs from Covid‐19 hospitalized patients may help identify prognostic circulating miRNAs, which could be useful to strategically improve clinical outcome.

Although we developed EV‐CATCHER, a targeted EV purification assay, for the fine‐tuned discovery of small‐RNA biomarkers associated with disease, we also ensured it allowed intact release of these small‐EVs for subsequent in vitro molecular analyses, which may not preclude in vivo analyses as well. As such, we chose to include an enzymatically degradable DNA linker (modified from Löf et al. 2017) between the capture antibody and our low non‐specific small‐RNA binding platform (Löf et al., 2017). We used TEM and nanoparticle tracking experiments to evaluate the intact release and enrichment of size‐specific small‐EV sub‐populations. Considering that circulating small‐EVs have been implicated in the immune response during viral infections (Bedford et al., 2020; Gu et al., 2020; Jeon et al., 2017; Urbanelli et al., 2019; Yao et al., 2018), with studies demonstrating that specific small‐EVs (exosomes) can harbour proteins that inhibit viral replication (e.g. against HIV (Khatua et al., 2009)) or contain proteins that can induce B lymphocyte proliferation (e.g., exosomes released from Epstein‐Barr Virus (EBV) infected cells (Gutzeit et al., 2014)), we tested the EV‐CATCHER assay to evaluate the potential role of circulating small‐EVs in viral neutralization of SARS‐CoV‐2. For these analyses, we used a second set of Covid‐19 serum samples, which included convalescent serum samples from Covid‐19 patients recovered from the SARS‐CoV‐2 infection (i.e., no active viral infection) and that was independent from the first sample set that was tested for small‐RNA sequencing of purified small‐EVs. Our initial experiments showed that highly‐purified small‐EVs obtained by successive ultracentrifugations, from the serum of patients containing high anti‐spike IgG titers, exhibited neutralizing activity against SARS‐CoV‐2 infection in vitro, whereas small‐EVs purified from the serum of patients with anti‐spike IgG titers below quantification level did not. We found that when using small‐EVs captured and released by the EV‐CATCHER assay from sera with high anti‐spike IgG titer, our in vitro analyses confirmed this small‐EV‐associated viral‐neutralizing activity, which again could not be observed with small‐EVs purified from sera with anti‐spike IgG titers below quantification level, or with small‐EVs purified by anti‐CD9/CD63/D81 EV‐CATCHER from patients at early stages of the SARS‐CoV‐2 infection (12 serum samples tested from the first set of Covid‐19 samples; Supplementary Figure 8). Several biological mechanisms may be at play to exert this neutralizing activity. Some groups, for example, have demonstrated that small‐EVs can transport or harbour immunoglobulins, which may explain the strong association between CD63^+^ small‐EV neutralization and the presence of high neutralizing anti‐spike IgG titers, however, no anti‐spike IgG was detectable by ELISA in our small‐EV purifications either by ultracentrifugation or with the anti‐CD63 EV‐CATCHER assay (Burkova et al., 2019; Huang et al., 2018). Additional mechanisms of viral neutralization have also been proposed, which could include an indirect cloaking strategy wherein convalescent circulating small‐EVs of patients, who elicited an immune response, may bind to cells targeted by the virus and prevent viral re‐entry (Gu et al., 2020), or a direct cloaking strategy where small‐EVs themselves may harbour host receptors for the viral spike protein (i.e. ACE2, the SARS‐CoV‐2 receptor) to cloak viral particles and prevent their cellular re‐entry (Burkova et al., 2019; Huang et al., 2018; Wang et al., 2020). Whereas the exact neutralizing mechanism of Covid‐19 convalescent circulating small‐EVs requires further evaluation, our observation that small‐EVs isolated from high anti‐spike IgG titer sera possess anti‐SARS‐CoV‐2 neutralizing activity has important implications for enhancement of convalescent plasma therapy or design of novel therapies. It is important to note that recent studies have demonstrated that immuno‐purified small‐EVs can maintain their functional properties (cellular uptake, signalling) with the purifying antibody attached to their surface (Chen et al., 2020; Hardin et al., 2018; Hisey et al., 2018; Hong et al., 2014; Patel et al., 2019; Peterson et al., 2015; Theodoraki et al., 2018; Velandia‐Romero et al., 2020). However, due to the possibility that some small‐EV functions may be compromised by antibody presence, for every new immuno‐purification protocol, it is necessary to initially evaluate their functionality by using small‐EVs purified by ultracentrifugation, as controls.

In summary, our proof‐of‐principle study demonstrates that in combination with a sensitive small‐RNA cDNA library preparation, our reproducible, customizable, and low non‐specific small‐RNA and small‐EV binding antibody‐based selection assay, EV‐CATCHER, can be successfully applied to the selective capture of small‐EVs. Interestingly, we found that our assay was generally more affordable (See Supplementary Table. 2) and that overall, it performed better than the majority of the 11 different small‐EV purification techniques assessed (displays low‐background, affords laboratory‐based customization, provides easy enzymatic release of intact small‐EVs) and more particularly it performed better than the 7 commercial magnetic bead‐based small‐EV purification kits. Based on its performance and sensitivity, EV‐CATCHER may help identify low‐expressed small‐RNAs encapsulated circulating biomarkers associated with disease, as suggested in previous small‐EV studies (Chen et al., 2018; Lai et al., 2018; Mathivanan et al., 2010; Vallabhajosyula et al., 2017). However, considering that the enzymatic release of the selected small‐EVs can only be performed once, due to remaining UNG activity present after elution, it may prevent use of a second antibody selection using EV‐CATCHER due to potential digestion of the uracylated DNA‐linker Thus, we propose that the addition of an initial ultrafiltration or ultracentrifugation step may provide an intact bulk isolation of small‐EVs from a sample, which may subsequently be subjected to antibody selection(s) by EV‐CATCHER. This initial ultrafiltration or ultracentrifugation step may also help decrease large‐sample volumes and thus prevent the use of multiple wells, where antibody‐capture is limited to 100–200μl samples per well. But importantly, the 96‐plex format of our assay, may allow streamlined automation for the purification and evaluation of diverse sub‐populations of circulating small‐EVs (based on the specific antibodies), with significant prospect for sequential small‐EV sub‐population capture and analysis from a single biofluid sample.

## CONFLICT OF INTERESTS

The authors declare that there is no conflict of interests regarding the publication of this paper.

## Supporting information

Supporting information.
