# Supplementary material for: Extracellular Vesicle Capture by AnTibody of CHoice and Enzymatic Release (EV‐CATCHER): A customizable purification assay designed for small‐RNA biomarker identification and evaluation of circulating small‐EVs
Source: J Extracell Vesicles. 2021 Jun 3;10(8):e12110. doi: 10.1002/jev2.12110 (PMC8173589; doi:10.1002/jev2.12110)
Supplement: Supplementary file 1 — Supporting information. [file JEV2-10-e12110-s001.pdf]

SUPPLEMENTARY FIGURE 1:

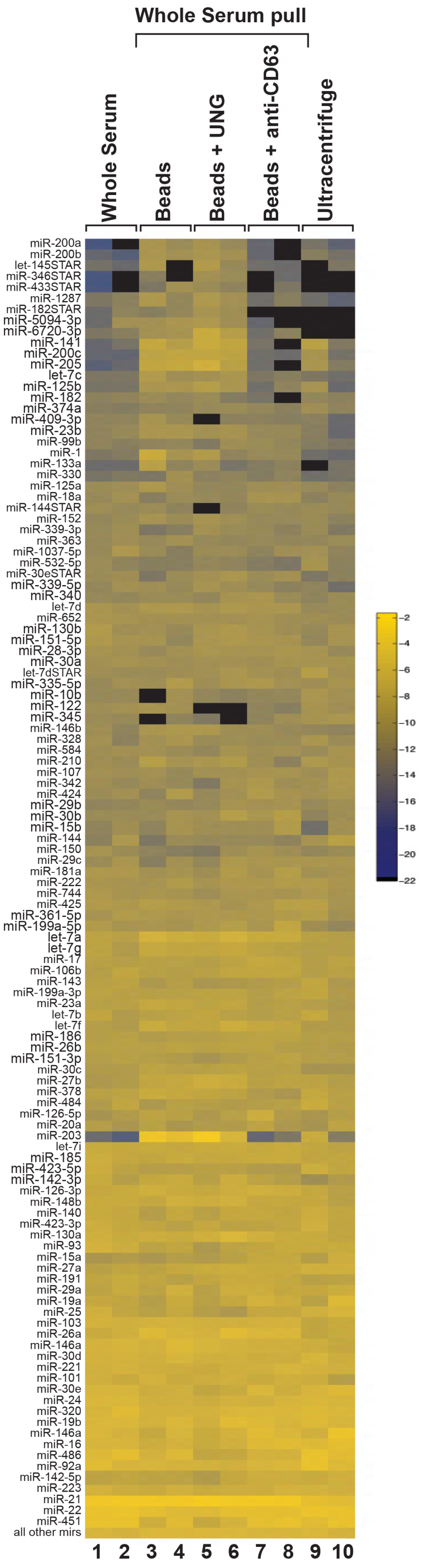

SUPPLEMENTARY FIGURE 2:

a.

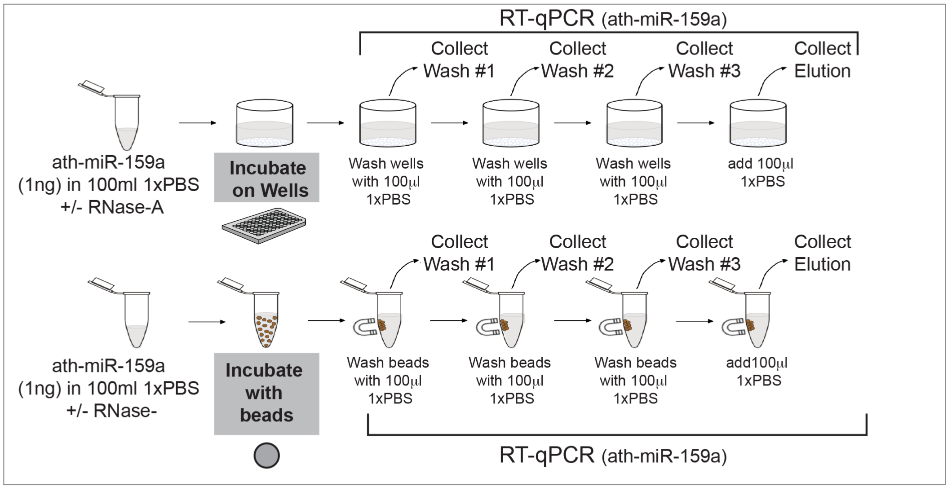

b.

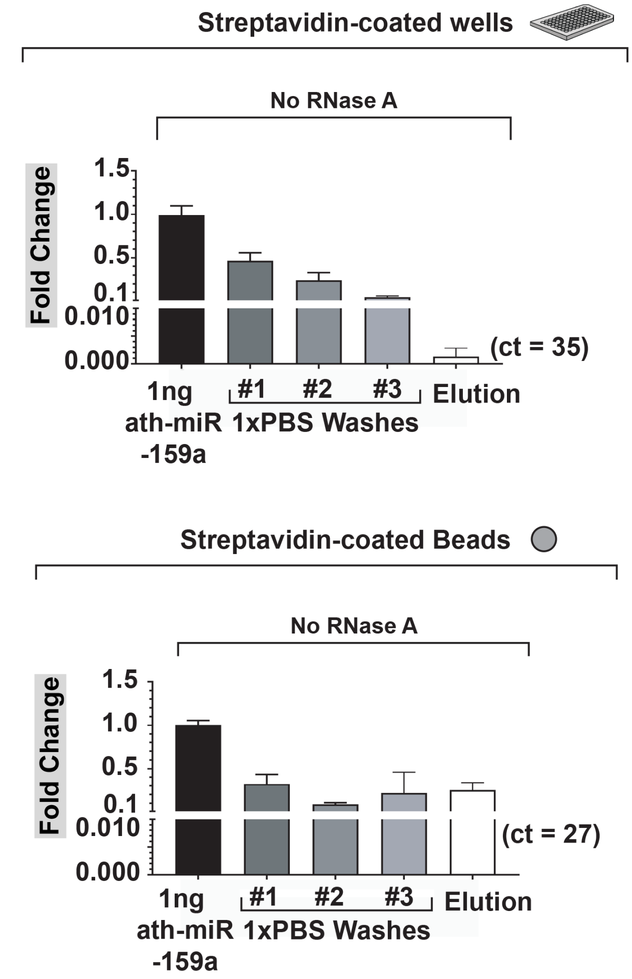

SUPPLEMENTARY FIGURE 3:

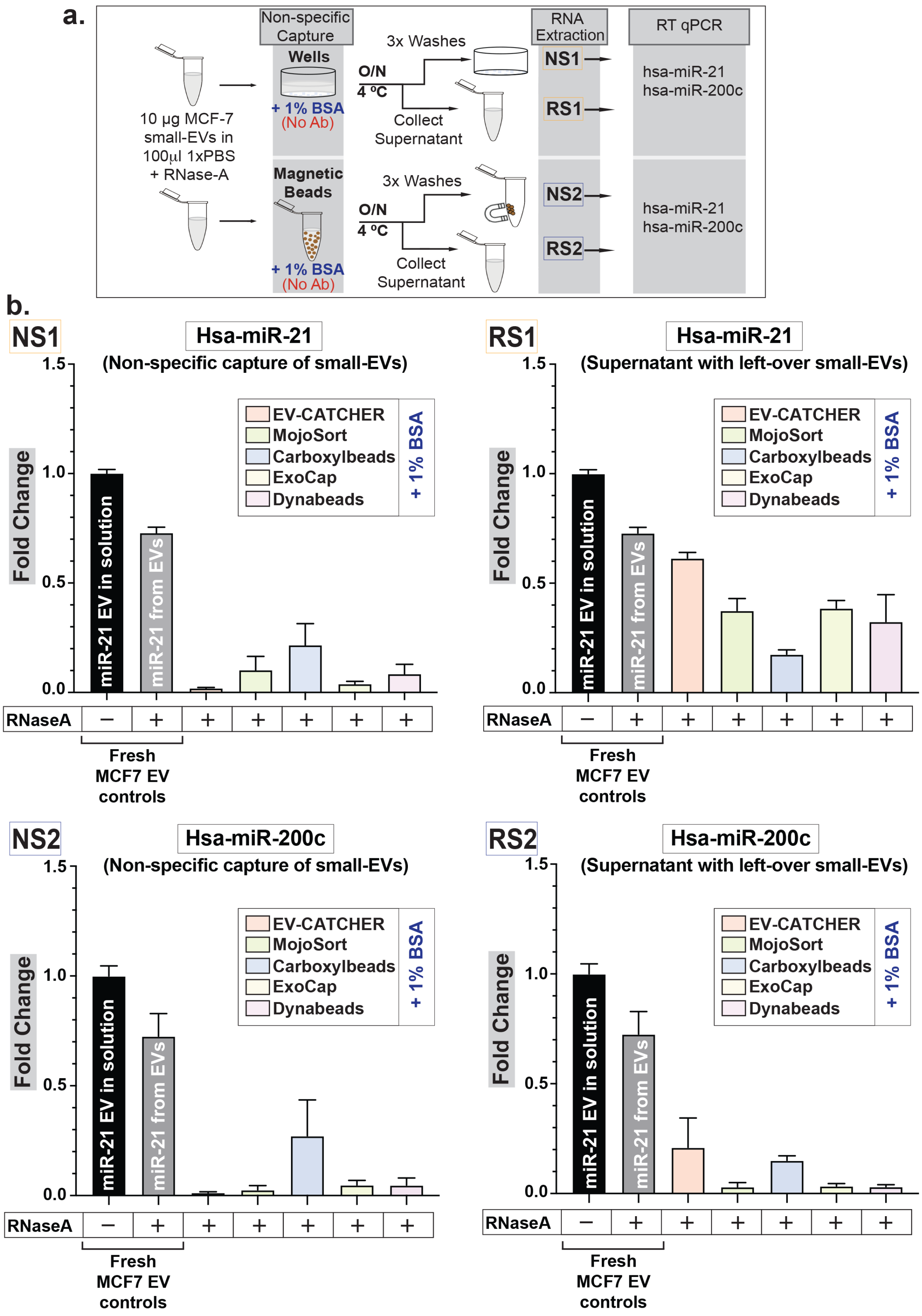

SUPPLEMENTARY FIGURE 4-

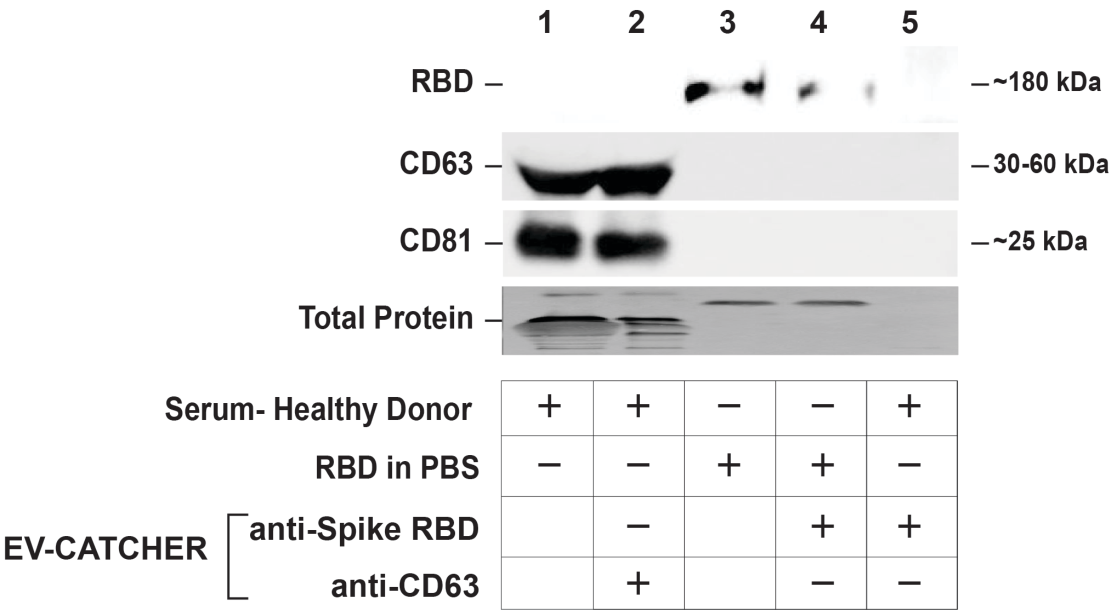

SUPPLEMENTARY FIGURE 5-

a.

Small-EVs ultracentrifuged from human plasma

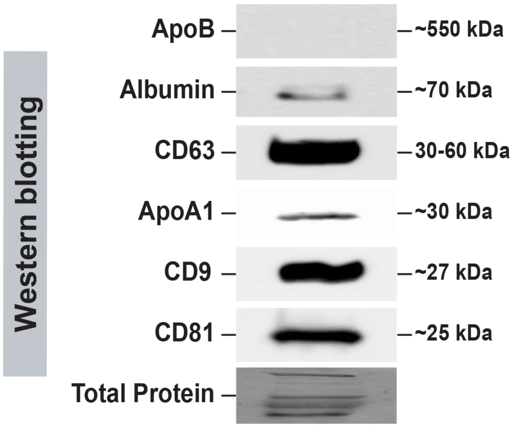

b.

Small-EVs ultracentrifuged from human plasma

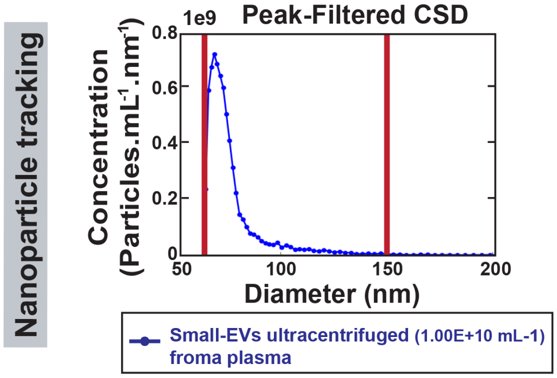

c.

Small-EVs ultracentrifuged from human plasma

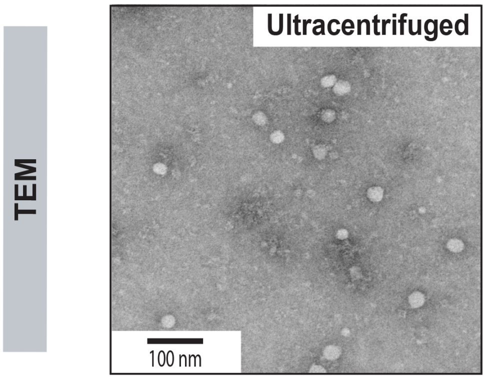

SUPPLEMENTARY FIGURE 6-

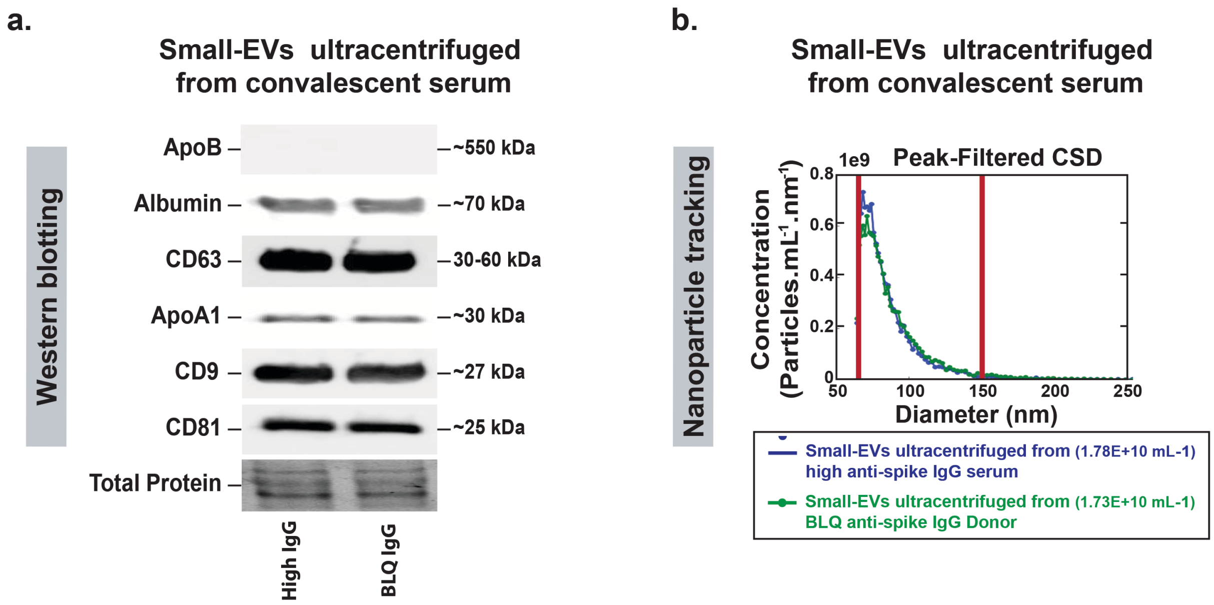

SUPPLEMENTARY TABLE 1:

Mild	Age	Gender	Assisted Ventilation	Duration of Hospitalization (days)	*Comorbidities	Severe	Age	Gender	Assisted Ventilation	Duration of Hospitalization (days)	*Comorbidities
**HUMC20**	27	F	No	11	Ash, DM, Endo, PCOS	**HUMC42**	63	M	Yes	17	HD, C, DM, GERD, HL, HTN, Ob, OSA, SOB
**HUMC23**	41	M	No	12	No PMHx	**HUMC44**	63	M	Yes	16	Ash, DM, ET, HT, HTN, OSA
**HUMC25**	54	F	No	2	DM, HL, HTN, UF	**HUMC75**	75	M	Yes	10	No PMHx
**HUMC28**	73	F	No	10	D, HTN, TD	**HUMC76**	58	M	Yes	21	No PMHx
**HUMC29**	57	M	No	10	No PMHx	**HUMC114**	54	M	Yes	29	No PMHx
**HUMC30**	87	M	No	10	C, BR, DVT, CVA, HL, HTN, Ob	**HUMC125**	57	M	Yes	22	C, HL, HTN
**HUMC34**	46	M	No	12	RFH, KD, DN, DM, ED, HM, HC, HTN	**HUMC309**	85	F	Yes	29	HD, DM, HC, GERD
**HUMC39**	64	M	No	7	HTN	**HUMC314**	76	M	Yes	8	CAD, HL, HTN, MFL, RLS
**HUMC41**	48	F	No	8	AN, CH, D, SOB, OSA, PN	**HUMC315**	75	M	Yes	19	HD, CHF, FR, HTN, VSU
**HUMC69**	60	M	No	2	D, DM, HTN, LTR	**HUMC324**	62	M	Yes	13	ACS, HTN
**HUMC127**	55	F	No	7	Ash	**HUMC326**	75	M	Yes	20	DM, HL, HTN
**HUMC130**	65	M	No	11	DM, GERD, HTN, OSA	**HUMC327**	69	M	Yes	14	GERD, HC
**HUMC43**	54	F	No	10	-	**HUMC328**	78	M	Yes	14	HTN
-	-	-	-	-	-	**HUMC342**	75	M	Yes	19	HTN
-	-	-	-	-	-	**HUMC37**	74	M	Yes	18	HTN
-	-	-	-	-	-	**HUMC123**	81	F	Yes	13	No PMHx
-	-	-	-	-	-	**HUMC24**	54	M	Yes	10	No PMHx

* Comorbidities are defined by the following lettering: Ash for Asthma, DM for diabetes melitus, Endo for endometriosis, PCOS for poly cystic ovary syndrome, PMHx for past medical history, HL for hyperlipidemia, HTN for hypertension, TD for thyroid disease, C for cancer, BR for bronchitis, DVT for deep vain thrombosis, CVA for cerebrovascular accident, Ob for obesity, RFH for respiratory failure with hypoxia, KD for kidney disease, DN for diabetic nephropathy, ED for erectile dysfunction, HM for heart murmur, HC for hypercholestrolemia, AN for anxiety, CH for chronic headaches, D for depression, SOB for shortness of breath, OSA for obstructive sleep apnea, PN for pneumonia, LTR for liver transplant recepient, GERD for gastroesophogeal reflux disease, HD for heart disease, ET for ear tumors, HT for high triglycerides, CAD for chronic artery disease, MFL for muscle function loss, RSL for restless leg syndrome, CHF for chongestive heart failure, FR for fractures, VSU for venous stasis ulcers, ACS for abnormal cardiovascular stress,

SUPPLEMENTARY FIGURE 7:

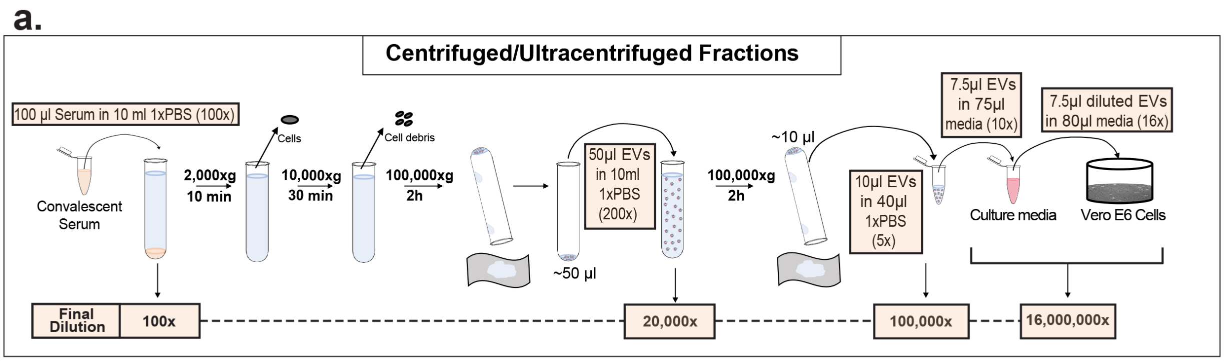

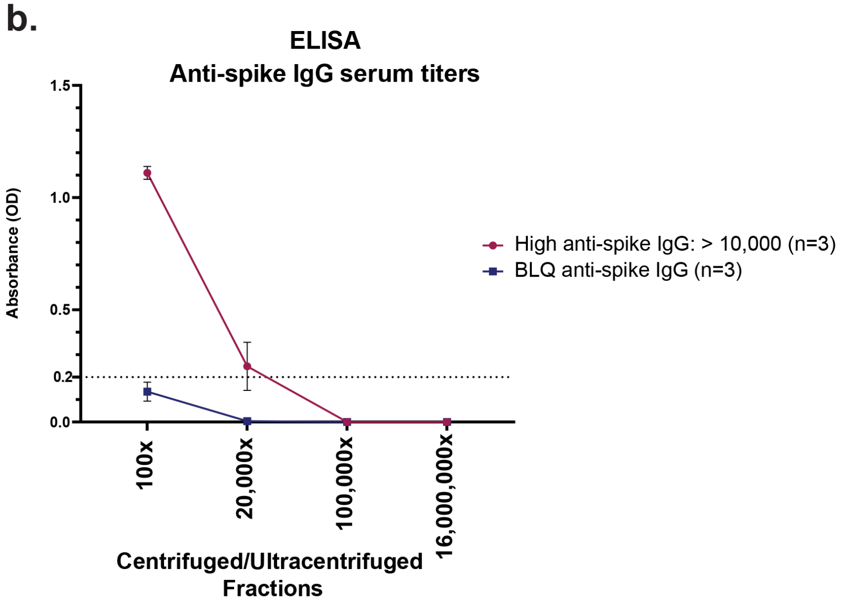

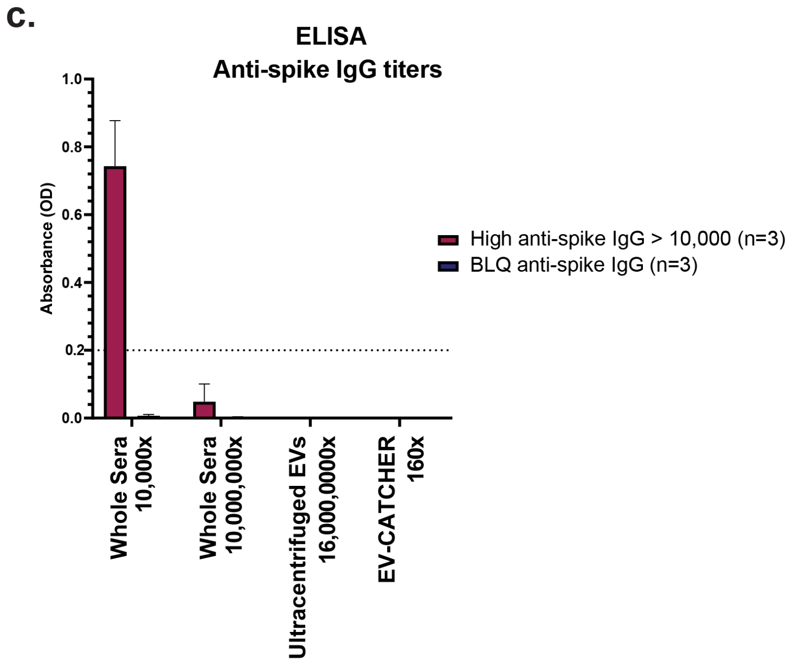

SUPPLEMENTARY FIGURE 8:

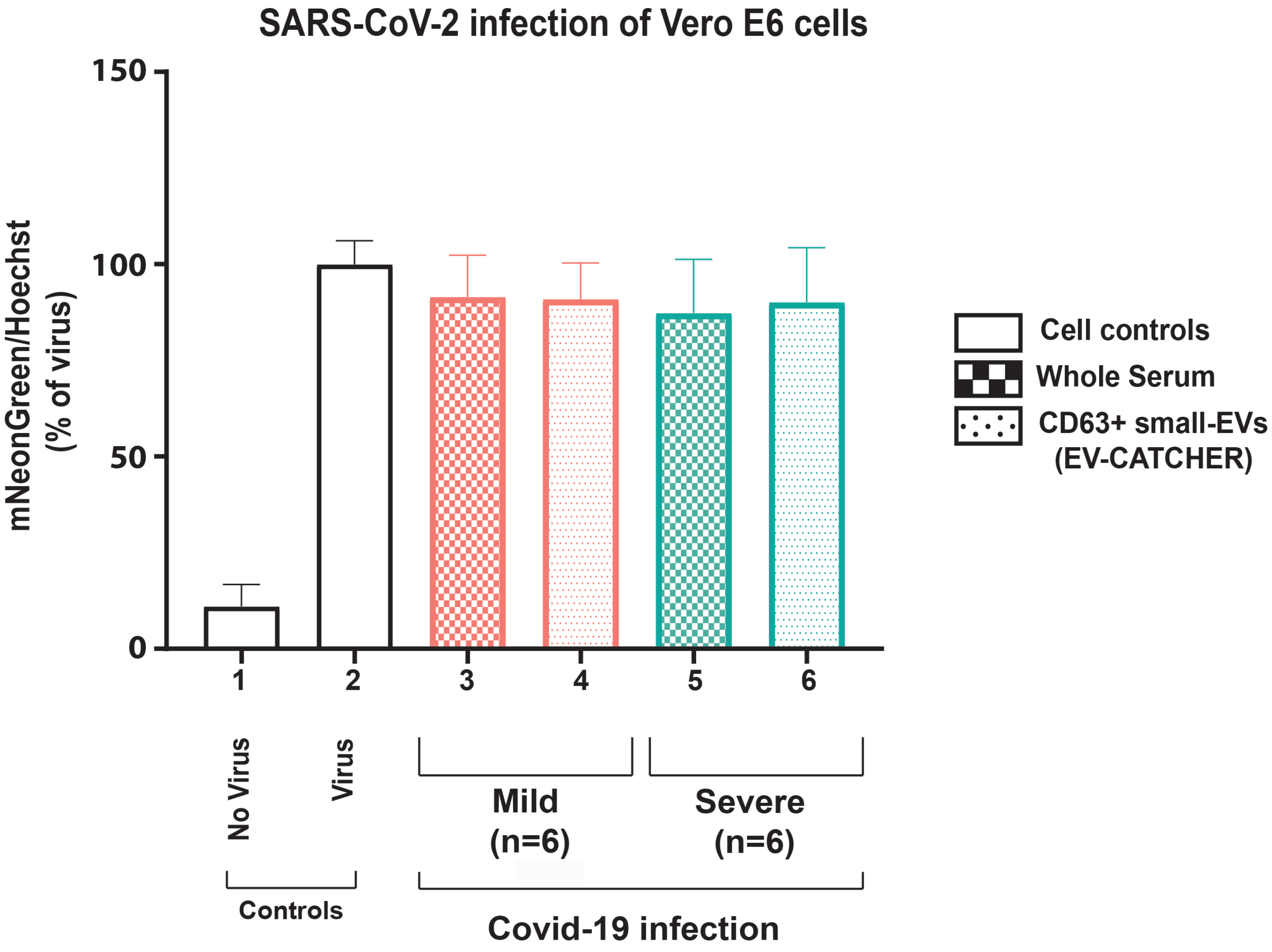

SUPPLEMENTARY TABLE 2:

	Diameter (µm)	Coated Height (µm)	Surface Area Formula	Surface Area Per Bead/Well	Volume Beads Per Reaction (µL)	Number of Beads Per Reaction	Total Surface Area Per Reaction	Cost per Reaction	Cost per Reaction with Ab
**EV-CATCHER**	6400	5500	$A = 2\pi rh + 2\pi r^2$	174920	N/A	N/A	1.75×10^5	\$0.53	\$6.24
**Miltenyi**	0.05	N/A	$A = 2\pi r^2$	7.85×10^{-3}	50	Proprietary	Proprietary	\$30.75	30.75
**MojoSort**	0.13	N/A	$A = 2\pi r^2$	0.053	10	Proprietary	Proprietary	\$2.50	\$6.85
**MagCapture**	1	N/A	$A = 2\pi r^2$	3.14	60	Proprietary	Proprietary	\$59.50	\$59.50
**Carboxyl**	1	N/A	$A = 2\pi r^2$	3.14	25	1.70×10^8	5.34×10^8	\$2.80	\$7.15
**ExoCap**	3	N/A	$A = 2\pi r^2$	28.27	100	1.20×10^7	3.39×10^8	\$26.47	\$30.82
**Dynabeads**	4.5	N/A	$A = 2\pi r^2$	63.62	100	1.70×10^6	1.08×10^8	\$12.93	\$12.93
**ExoFlow**	9.1	N/A	$A = 2\pi r^2$	260.16	20	3.2×10^5	8.33×10^7	\$17.56	\$17.56

FIGURE LEGENDS – SUPPLEMENTARY DATA

Supplementary Figure 1- Small-RNA sequencing of exosomes purified from serum using magnetic beads. Heatmap representation of the top 117 expressed circulating small-RNAs in human serum identified by small-RNA next-generation sequencing (columns 1 and 2). Small-RNA sequencing was performed on RNA extracted from Dynabeads magnetic beads incubated in whole serum (columns 3 and 4), magnetic beads incubated with whole serum and treated with UNG (columns 5 and 6), magnetic beads covalently bound to CD63 antibody incubated in whole serum (columns 7 and 8) compared to exosomes obtained by ultracentrifugation (columns 9 and 10). All experiments were performed in duplicate.

Supplementary Figure 2- RT-qPCR evaluation of non-specific miRNA binding to streptavidin-coated wells and magnetic beads through three washes and a final elution- a. Schematic representation for incubation of synthetic ath-miR-159a miRNA (1ng in 100μl 1xPBS) with streptavidin coated wells and Dynabeads MyOne^{TM} T1 streptavidin-coated magnetic beads followed by three individual 1xPBS washes and a final 1xPBS elution. **b.** RT qPCR detection of ath-miR-159a in the initial solution (1ng ath-miR-159a), after each individual 1xPBS washes (#1, #2, and #3) and a final 1xPBS elution for streptavidin coated wells (top graph), and with streptavidin-coated magnetic beads (Dynabeads) (bottom graph). All RT-qPCR reactions were performed in triplicate and the expression fold change between the individual experiments and the control solution (1ng) were evaluated using the $2^{\Delta\Delta Ct}$ formula.

Supplementary Figure 3- RT-qPCR evaluation for non-specific binding of small-EVs to streptavidin-coated wells and four different customizable magnetic beads- a. schematic describing evaluation of the non-specific binding of small-EVs using MCF7 small-EVs/exosomes (SBI; 10μg) to streptavidin-coated wells and four different commercially available magnetic beads (3 streptavidin-coated and 1 carboxyl-terminated) in presence of 1%BSA but without a conjugated antibody. Non-specific binding of small-EVs was evaluated by RT-qPCR quantification of hsa-miR-21 and hsa-miR200c

Remaining in Solution (RS) after incubation with wells (top; RS1) or magnetic beads (bottom; RS2), or Non-Specifically (NS) bound, after three 1xPBS washes, to the wells (NS1) or to the magnetic beads (NS2). **b.** Graphs displaying RT-qPCR data for non-specific binding of small-EV by quantification of hsa-miR-21 (top two graphs) and hsa-miR200c (bottom two graphs) after incubation with streptavidin-coated wells or magnetic beads. For all graphs the black bars display total detection of hsa-miR-21 (top two graphs) and hsa-miR200c (bottom two graphs) within 10 μg of MCF7 small-EVs, and the grey bars display detection of hsa-miR-21 (top two graphs) and hsa-miR200c (bottom two graphs) after RNase-A treatment and removal of free-floating miRNAs. The top two graphs display quantification of hsa-miR-21 between wells and magnetic beads for evaluation of non-specifically bound small-EVs (left) or for measure of small-EVs remaining in solution (right), after processing 10μg MCF7 small-EVs/exosomes in presence of RNase-A. The bottom two graphs display the RT-qPCR detection of hsa-miR-200c between wells and magnetic beads for evaluation of non-specifically bound small-EVs (left) or for measure of small-EVs remaining in solution (right), after processing a solution of 10μg MCF7 small-EVs/exosomes in presence of RNase-A. All RT-qPCR reactions were performed in triplicate and the expression fold change between the experiments and the control solution were evaluated using the $2^{\Delta\Delta Ct}$ formula.

Supplementary Figure 4- Evaluating specificity of the EV-CATCHER assay - Western blot analyses of small-EV surface proteins CD63 and CD81 from whole human serum (Lane 1) and from small-EVs purified from whole human serum using the anti-CD63 EV-CATCHER assay (Lane 2). Western blot analyses of the SARS-CoV-2 receptor binding domain (RBD) protein (provided by Dr. Perlin's laboratory), as a concentrate (2μg in Lane 3) or after anti-RBD EV-CATCHER purification (combined purification of 2 wells with each 1 μg in 100 μl 1xPBS) (Lane 4). Lane 5 displays the proteins purified from healthy whole human serum (purchased from Sigma (Cat#H4522) before Covid-19 pandemic) after anti-RBD EV-CATCHER purification. The total protein evaluation indicates absence of non-specifically purified small-EVs serum (no detection of CD63 or CD9 proteins).

Supplementary Figure 5- Evaluation of small-EVs ultracentrifuged from human plasma- a. Western blot analysis of ApoB, Albumin, CD63, ApoA1, CD9, and CD81 proteins after small-EVs purification by ultracentrifugation from human plasma. **b.** Spectradyne nanoparticle tracking using a TS400 microfluidic cartridge for quantification of small-EVs ultracentrifuged from human plasma and size evaluation of the sedimented small EVs that display sizes ranging between 60-150nm. **c.** Transmission Electron Microscopy (TEM) of small-EVs purified from human plasma by ultracentrifugation.

Supplementary Table 1- Descriptive characteristics of Covid-19 sera samples. Clinical data on Covid-19 serum samples collected at Hackensack University Medical Center (HUMC). Clinical parameters of Covid-19 hospitalized patients, including age, gender, duration of hospitalization, assisted ventilation status and known comorbidities. Mild cases were designated as patients positive for Covid-19 who were hospitalized but did not receive mechanical ventilation (n=13) and severely ill cases were designated as patients with ARDS requiring mechanical ventilation (n=17).

Supplementary Figure 6- Evaluation of small-EVs ultracentrifuged from convalescent serum from high and below quantification anti-spike IgG antibodies- a. Western blot analysis of ApoB, Albumin, CD63, ApoA1, CD9, and CD81 after ultracentrifugation of small-EVs from high anti-spike IgG (left column) and below level of quantification anti-spike IgG (BLQ, right) human SARS-CoV-2 convalescent sera. **b.** Spectradyne nanoparticle tracking using a TS400 microfluidic cartridge to evaluate quantity and size of small-EVs ultracentrifuged from high and BLQ anti-spike IgG human SARS-CoV-2 convalescent sera.

Supplementary Figure 7- ELISA quantification of anti-spike (RBD region) IgG from small-EVs ultracentrifuged and anti-CD63 EV-CATCHER purified from convalescent sera. a. Schematic representation of the centrifugation and ultracentrifugation steps taken to sediment small-EVs from three high anti-spike IgG and three below level of quantification (BLQ) anti-spike IgG SARS-CoV-2 human convalescent sera. Details on the dilutions with 1xPBS solutions are described in the

yellow rectangles above the centrifugation glass tubes. The final dilutions for each of the 1xPBS resuspension are detailed in the bottom yellow rectangles, with an estimated final 16,000,000x dilution. **b.** ELISA quantification of anti-spike (RBD region) IgG titers from three high anti-spike IgG (red) and three BLQ (Blue) anti-spike IgG SARS-CoV-2 human convalescent sera after estimated dilutions of 100x, 20,000x, 100,000x, and ~16,000,000x. **c.** ELISA detection of anti-spike (RBD region) IgG in 10,000x diluted (standard for detection of IgG activity), in 10,000,000x diluted, ultracentrifuged (16,000,000x diluted), and EV-CATCHER anti-CD63 purification before and after and 160x dilution, from three high (red) and three BLQ (blue) anti-spike IgG SARS-CoV-2 human convalescent sera. All experiments and measures were performed in triplicate, hence the error bars.

Supplementary Figure 8- Evaluating SARS-CoV2 neutralizing properties of whole serum and small-EVs purified with the anti-CD63 EV-CATCHER assay from the sera of SARS-CoV-2 mildly and severely ill hospitalized patients. Vero E6 cells were treated with whole serum (squared checked bars, 3 and 5) and small-EVs purified by the anti-CD63 EV-CATCHER assay (dotted bars, 4 and 6) from the sera of 6 mildly ill (no mechanical ventilation) and 6 severely ill (mechanical ventilation) patients who were hospitalized at HMH, prior to *in vitro* infection with the SARS-CoV-2 mNeonGreen virus. The cell controls display the fluorescence detectable from untreated/uninfected cells (bar #1) and SARS-CoV-2 mNeonGreen infected VeroE6 cells (bar #2). Serum samples were obtained at the time of hospitalization.

Supplementary Table 2- Surface area and cost comparison between customizable streptavidin-coated wells and four types of customizable magnetic beads (Streptavidin-coated or Carboxyl-terminated beads). Using the magnetic-bead diameter provided by the manufacturers, the total surface of the beads for manufacturer recommended volume of beads (individual reactions) was calculated. Surfaces provided on magnetic beads exceeds surface from individual 96 well-plate streptavidin coated wells. Total cost per reaction was evaluated with EV-CATCHER offering the cheapest option.
